# New Vistas on the Anionic Polymerization of Styrene in Non-Polar Solvents by Means of Density Functional Theory

**DOI:** 10.3390/polym8100371

**Published:** 2016-10-20

**Authors:** Hideo Morita, Marcel Van Beylen

**Affiliations:** 1 Honorary consultant for Synthetic Rubber Division, Asahi Kasei Corp., Torigaoka 50-5, Totsuka-ku, Yokohama 244-0001, Japan; 2 Professor Emeritus, Laboratory of Macromolecular and Physical Organic Chemistry, Department of Chemistry, Catholic University of Leuven, Celestijnenlaan 200F, Heverlee (Leuven) B-3001, Belgium; marcel.vanbeylen@chem.kuleuven.be

**Keywords:** anionic polymerization, DFT calculation, M062X, non-polar solvent, penultimate unit effect, polymerization mechanism, steric hindrance, styrene, transition state

## Abstract

The elementary processes of anionic styrene polymerization in the gas phase and in cyclohexane were studied using M062X (a recently developed density functional theory (DFT) method) combined with the 6-31+G(d) basis sets, in order to clarify the complicated phenomena caused by the association of the active chain-ends and elucidate the details of the polymerization mechanism. Three types of HSt_2_Li (a model structure of polystyryllithium chain-ends) were obtained; the well-known first structure in which Li is coordinated to the side chain, the second structure in which Li is coordinated to the phenyl ring, (both without the penultimate unit coordination), and the third structure in which Li is coordinated to both the chain-end unit and the penultimate styrene unit. Although the third HSt_2_Li is the most stable as expected, the free energy for the transition state of its reaction with styrene is higher than those for the other two transition states due to its steric hindrance. The free energy for the transition state of the reaction of the second HSt_2_Li with styrene is the lowest, suggesting that the route through it is the predominant reaction path. The penultimate unit effect, slower addition of styrene to HSt_2_Li than to HStLi, is attributed to coordination of the penultimate styrene units of the polystyryllithium dimer (one of the starting materials) to its Li atoms. The calculated enthalpy for the reaction barrier of the second HSt_2_Li with styrene in cyclohexane was found to agree with the observed apparent activation energy in benzene.

## 1. Introduction

Anionic polymerization of vinyl monomers such as dienes and styrene is currently the best known and most controllable of all types of polymerization. Its course and mechanism, studied by M. Szwarc [1], S. Bywater, and other workers in the field, have now been elucidated into their detailed intricacies [2,3,4,5]. For the anionic polymerization of styrene in non-polar solvents, it is generally accepted that polystyryllithium (PStLi) is mainly associated into dimers (PStLi)_2_ in equilibrium with a small amount of non-associated PStLi chains. A kinetic order of 0.5 with respect to [PStLi] indicates that only monomeric (non-associated) PStLi ion-pairs are able to propagate [6,7]. However, a possibility of the reaction of dimeric (PStLi)_2_ and higher aggregates with styrene was suggested on the basis of the experimental data such as addition of butadiene to freeze-dried polystyryllithium and existence of the higher aggregates demonstrated by means of light scattering and small angle neutron scattering [8,9]. Counter arguments were presented against their assertion [10], and no decisive evidence for the advantage of polymerization of the dimeric species has been shown as far as we know.

Theoretical calculations by means of quantum-chemical methods were mainly reported on investigation of the aggregated states of organolithium compounds using MOPAC, ab initio and density functional theory (DFT) methods, and considerable contributions were made in the field [11,12,13]. Theoretical approaches to the mechanism of anionic polymerization of dienes [14,15,16] and copolymerization of styrene and butadiene [17] were also reported. However no theoretical report has been found on the mechanism of styrene polymerization.

Theoretical calculations, in general, not only support the experimentally established reaction mechanism but also cover unavoidable weak points of experiments such as poor information on substances with short life time (e.g., structures of transition states) and on substances existing in very low concentrations (active polystyryllithium species, in our case). Also, the recent progress on the development of DFT methods is remarkable, and calculated data obtained using the new DFT methods are in better agreement with the experimental results than those using the old ones [18,19]. The purpose of this study is to elucidate in detail the mechanism of styrene anionic polymerization with organolithium initiators in non-polar solvents by means of a recently developed theoretical method. In this paper the geometries of the dimeric and monomeric species and dissociation enthalpies and free energies of the first into the second are calculated, and the pathways of the reaction of addition of styrene to the small proportion of monomeric polystyryllithium are also shown using M062X, a recently developed DFT method. The results not only support the mechanism established by experiments but also answer the following questions which cannot be answered experimentally.

(a) What is the structure of the predominant transition state of the reaction? Is it the same as the experimentally expected one?
(b) Does the most stable polystyryllithium active species govern the reaction? If not, why?
(c) What is the penultimate unit effect (slower addition of styrene to HSt_2_Li than to HStLi, [20,21]) due to? Is it reasonably explained?

Thus, a deep insight into the structural phenomena of the atoms in the reacting molecules is provided, and the whole observed mechanism becomes evident and clearly understood.

In this paper, the reaction of monomeric polystyryllithium is fully reported. For the reaction of dimeric polystyryllithium a preliminary result is shown in this report, and the detailed study will be reported in our next paper in the near future.

## 2. Methods

Polystyryllithium species that are obtained by the addition of styrene to alkyllithium can be expressed as R(St)_m_Li, where *R* denotes the alkyl group of the initiator. We employ HStLi and HSt_2_Li by setting *m* = 1 and 2, and substituting H for R. With these structural models, the dissociation of dimeric polystyryllithiium into monomeric polystyryllithium and the addition of styrene to the non-associated polystyryllithium (a propagation reaction) are studied.

Preliminary calculations of the anionic homopolymerization of styrene and butadiene were performed using several quantum-chemical calculation methods including ab initio MP2 [22,23,24], B3LYP [25] (the widely used DFT method), and M062X [19] (a recently developed DFT method). The obtained reaction barriers of styrene and butadiene were compared with the experimentally observed apparent activation energies of their homopolymerization, the M062X method was selected, and the calculations of this study were performed using the M062x/6-31+G(d)//M062x/6-31+G(d) method with the Gaussian 09W program [26]. The detailed results on the calculation methods will be reported in our paper on the anionic homo- polymerization of butadiene in the near future. The optimization of active chain-ends (HSt_m_Li (*m* = 1 or 2)), their dimers (HSt_m_Li)_2_, and the intermediates and products of the reaction of HSt_m_Li with styrene (St/HSt_m_Li; the precursor complexes, the transition states, and the products) were performed, and the obtained geometries and values of enthalpy and Gibbs free energy at 25 °C were used in the discussion. The transition states were confirmed to have one imaginary frequency. The precursor complexes and the products were obtained by first applying the IRC method [27,28,29] to the transition states, then optimizing the obtained intermediate structures completely. For calculations in cyclohexane and THF (tetrahydrofuran), the polarizable continuum model (PCM) [30,31], a widely used method, was employed.

In order to compare the stability of the structures in the gas phase, the values of relative enthalpy in the gas phase (∆*Hr)* were calculated based on the obtained values of enthalpy in Table A1, according to the following procedure:

(a) For (HSt_m_Li)_2_ (*m* = 1 or 2),
∆*Hr* = *H*[(HSt_m_Li)_2_] − *H*[(HSt_m_Li)_2_]_0_
  where *H*[(HSt_m_Li)_2_] denotes the enthalpy of the particular (HSt_m_Li)_2_, and *H*[(HSt_m_Li)_2_]_0_ is the enthalpy of (HSt_m_Li)_2_ with the lowest free energy of the studied dimers, for which ∆*Hr* = 0. In this case, ∆*Hr* indicates the extent of instability (in a thermodynamic sense) of the particular dimer, (HSt_m_Li)_2_, with respect to the dimer with the lowest free energy.
(b) For HSt_m_Li,
∆*Hr* = *H*(HSt_m_Li) − 1/2*H*[(HSt_m_Li)_2_]_0_
  where *H*(HSt_m_Li) denotes the enthalpy of the particular HSt_m_Li. In this case, ∆*Hr* indicates the dissociation energy of (HSt_m_Li)_2_ with the lowest free energy into the particular HSt_m_Li per HSt_m_Li molecule.
(c) For St/HSt_m_Li (precursor complexes, transition states, or products).
∆*Hr* = *H*[(St/HSt_m_Li)] − [*H*(St) + 1/2*H*[(HSt_m_Li)_2_]_0_]
  where *H*[(St/HSt_m_Li)] denotes the enthalpy of the particular St/HSt_m_Li system and *H*(St) is the enthalpy of styrene. In this case, ∆*Hr* indicates the extent of instability of the particular St/HSt_m_Li system with respect to the starting material (styrene and the dimeric (HSt_m_Li)_2_). ∆*Hr* for the transition state corresponds to the apparent activation energy. (The relative energies are calculated with respect to the starting materials, styrene and (HSt_m_Li)_2_ in this case. The reaction between one styrene molecule and one HSt_m_Li molecule (a half (HSt_m_Li)_2_ molecule) is treated here. Therefore, ∆*Hr* is calculated with respect to (St + 1/2(HSt_m_Li)_2_).

The values of the relative free energy in the gas phase (∆*Gr)* were also calculated in the same way as those for ∆*Hr*, using the values of *G* in Table A1 instead of *H*.

∆*Hrch* and ∆*Grch*, the values of relative enthalpy and relative free energy in cyclohexane, were calculated in similar ways to ∆*Hr* and ∆*Gr* in the gas phase using the values of *H* and *G* in cyclohexane shown in Table A2. ∆*Hrth* and ∆*Grth,* the values of relative enthalpy and relative free energy in THF, were calculated for the monomeric and dimeric polystyryllithium in similar ways to ∆*Hr* and ∆*Gr* using the values of *H* and *G* in THF shown in Table A3.

For some structures, basis set superposition errors (BSSE) were evaluated using the counterpoise method [32]. The results are shown in Appendix A2.

In all geometries of the studied structures, C–Li distances less than 0.245 nm were marked with full or dotted lines as η-coordinated bonds.

## 3. Results and Discussion

### 3.1. (HStLi)_2_, HStLi, and the Addition of Styrene to HStLi in the Gas Phase

#### 3.1.1. (HStLi)_2_ and HStLi

**(HStLi)_2_**. While dimers of alkyllithium are known to contain 4-membered cycles formed by two C–Li bonds [11,12,13], structures of dimers of aromatic lithium compounds like (HStLi)_2_ were not known well. New structures, in which the side chain of each HStLi is coordinated to one lithium atom and the phenyl ring to the other lithium atom, have recently been suggested based on DFT calculations by A. Yakimansky and M. Van Beylen (one of the authers) [33]. Two chain-end units (HSt-) surround the two lithium atoms, and the structures were referred to as ‘sandwich’ structures. (Reference [33] will be referred to as Paper I hereafter).

The optimization of possible model dimer structures, (HStLi)_2_, was performed, and the obtained structures are shown in Figure 1-1 ((1-a) through (1-f)). In the figure, C–Li distances less than 0.225 nm are shown. In (1-a) and (1-b), each Li is η^1^-coordinated to the α-carbon of the side chain of one HStLi and η^6^-coordinated to the phenyl ring of the other HStLi. The Li–Li distances are ca. 0.33 nm. The geometries for (1-a) and (1-b) are different in the direction of the side chains, in the same or opposite direction to the two Li atoms, and their symmetries are C_i_ and C_2_. In (1-c) and (1-d), one Li is η^1^-coordinated to the α-carbon of the side chain of each HStLi, and the other Li is η^4^-coordinated to the phenyl ring of each HStLi, and their Li–Li distances are ca. 0.29–0.30 nm. The geometries for (1-c) and (1-d) are different in the direction of the side chains, in the same or opposite direction to the two Li atoms, (1-c) being C_2_-symmetric and (1-d) being asymmetric. The geometries for (1-b) and (1-c) are shown in Paper I as Figure 1 (b) and (c).

C–C bond distances of (1-a) and (1-c) are shown in Figure 1-2 as (1-g) and (1-h). Since the C–C bond distances of (1-b) are essentially the same as those of (1-a) (which is shown in (1-g)), the C–C bond distances of (1-b) are not shown here (the differences are ≤0.001 nm). The C–C bond distances of (1-d) are also the same as those of (1-c) (which is shown in (1-h)), and are not shown. In the geometries for (1-g) (= (1-a)) and (1-h) (= (1-c)), the (α)C–(ipso)C bond distances of the side chains are 0.140–0.141 nm, indicating double bond character. This suggests that the π-electron density is not only delocalized at the phenyl ring, but also is extended to the (α)C–(ipso)C bond of the side chain.

In (1-e) and (1-f), each Li atom is η^1^-coordinated to the α-carbon of the side chain of each HStLi and η^3^- or η^2^-coordinated to the side chain of one HStLi centered on the ipso-carbon. The Li–Li distances are ca. 0.24 nm, which is close to that of the methyllithium dimer calculated at the same M062X/6-31+G(d)//M062X/6-31+G(d) level (ca. 0.22 nm). The two phenyl rings are apart from the two Li atoms and arranged on opposite sides. The structures (1-e) and (1-f) are different in the direction of the side chains and their structures are not symmetrical. Their (α)C–(ipso)C bonds are 0.145–0.146 nm, suggesting double bond character. At first sight, they look like structures with 4-membered cycles formed by two C–Li bonds. In reality, they look more like structures with distorted 6-membered cycles formed by two (α)C–Li half-bonds, two (ipso)C–Li half-bonds, and two (α)C–(ipso)C bonds (taking the four shortest C–Li bonds into account). The optimization failed for the 4- or 6-membered-cycle structure in which both phenyl rings were located on one side of the two Li atoms.

Structure (1-a) has the lowest free energy of the studied (HStLi)_2_ dimers, as shown in Table A1. Accordingly, the values of relative enthalpy (∆*Hr*) and relative free energy (∆*Gr*) for the other dimers were calculated with respect to the enthalpy and free energy of (HStLi)_2_(1-a) (*H*[(HStLi)_2_]_0_ and *G*[(HStLi)_2_]_0_), for which ∆*Hr* or ∆*Gr* = 0, and are shown in the parentheses in Figure 1-1. Energetically (1-a) and (1-b) are essentially equal, although ∆*G* for (1-a) is slightly lower. ∆*Hr* and ∆*Gr* for (1-c) and (1-d) are higher than those for (1-a) and (1-b) by ca. 10 kJ·mol^–1^, indicating that the latter two structures are more stable. ∆*Hr* and ∆*Gr* for (1-e) and (1-f) are higher than those for (1-a) and (1-b) by about 50 kJ·mol^–1^, indicating that (1-a) and (1-b) are much more stable than (1-e) and (1-f). This result suggests a stronger interaction between the phenyl ring and Li than between the side chain and Li. Since (1-a) has the lowest free energy, as discussed above, this structure and its relative energies represent those of (HStLi)_2_ hereafter. (The energetic values for the whole dimer system should be energetically weighted and averaged for all related dimers, strictly speaking. However, the concentration of the dimer with the lowest free energy (as determined by the free energy difference from the other dimers) is higher than the concentration of the other dimers, and its energetic values are close to the average energetic values of the whole system. Therefore, the energetic values of the dimer with the lowest free energy will be used to represent those of the whole system).

**HStLi.** The widely accepted structure of the non-associated polystyryllithium chain-end (PStLi) in non-polar solvents is the structure in which Li is bound to the α-carbon of the side chain, P’–CH_2_–CHLi–Ph [4], which is the same as that in polar solvents. It may have been readily accepted since the polymerization proceeds through the reaction of the side chain double bond of styrene. However, no experimental evidence for this structure has been shown to our knowledge. Since there is only a low proportion of non-associated PStLi that is in equilibrium with a large amount of dimeric (PStLi)_2_ in non-polar solvents, characterization of the non-associated PStLi by experimental methods is difficult. Some quantum-chemical approaches for benzyllithium were reported [34,35]. In these papers two structures of benzyllithium, one in which Li is coordinated to the side chain, the ‘classical’ structure, and the other in which Li is coordinated to the phenyl ring, are shown.

The optimization of possible structures of HStLi was performed and the obtained structures are shown in Figure 2-1 as (2-a) through (2-c). The Li atoms of (2-a) and (2-b) are η^3^-coordinated to the side chains (to (α)C, (ipso)C, and (ortho)C, to be exact). Structures (2-a) and (2-b) differ based on the position of Li relative to the side chain, i.e., outside or inside the side chain. (2-a) is also shown in Paper I as Figure 1 (a). The Li atom of (2-c) is η^6^-coordinated to the phenyl ring. ∆*Hr* and ∆*Gr* for these structures, the enthalpies and free energies of the dissociation of (HStLi)_2_(1-a) into the particular HStLi per HStL molecule, are shown in the figure. ∆*Hr* for (2-a) and (2-b) are almost the same, and ∆*G* for (2-a) and (2-b) are also almost the same. However, ∆*Hr* and ∆*Gr* for (2-c) are lower than those for (2-a) and (2-b) by 6–7 kJ·mol^–1^, indicating that (2-c) is more stable than (2-a) and (2-b). A stronger interaction between the phenyl ring and Li than between the side chain and Li is shown again. The energetic data for (2-a) through (2-c) suggest that the dissociation of (HStLi)_2_(1-a) into HStLi requires a large energy as shown below.

1/2(HStLi)_2_(1-a) → HStLi(2-a), (2-b), or (2-c) (∆*Hr =* 107–113 kJ·mol^–1^, ∆*Gr* = 82–89 kJ·mol^–1^)

Therefore, in the case where only HStLi and (HStLi)_2_ are present in the system, e.g., when polymerization is completed, the dissociation needs such high energies that only a low proportion of HStLi is dissociated. However, when polymerization is proceeding in the presence of styrene, HStLi will complex with styrene in the course of dissociation or before dissociation, and so large amounts of dissociation energy will not be needed. This process will be discussed later in detail.

It is worth noting that the (α)C-(ipso)C bond distances of (2-a) and (2-b) are 0.143 and 0.141 nm, respectively, indicating double bond character. Their Li atoms are η^3^-coordinated, and the distances of the (ipso)C-Li bonds are nearly the same as those of the (α)C–Li bonds (0.208 vs. 0.205 nm for (2-a) and 0.207 vs. 0.202 nm for (2-b)). In Figure 2-2 distribution of the electron density of HStLi (2-a) is shown. The figure clearly shows that the electron density is not only delocalized at the phenyl ring, but it is also extended to the (α)C–(ipso)C bond, forming an η^3^-coordination of Li with the side chain, like the well-known bridged structure of allyllithium [36,37].

**Figure 3:**
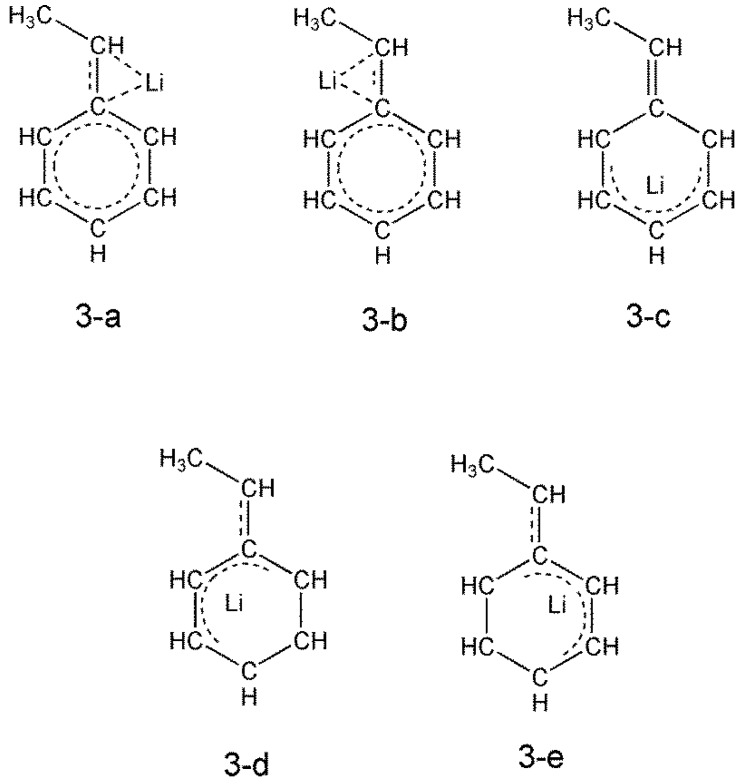
Schematic structural formulae of HStLi.

Structures (3-a), (3-b), and (3-c) in Figure 3 are the schematic structural formulae of (2-a), (2-b), and (2-c) in Figure 2-1, respectively, formulated based on the bond lengths shown in Figure 2-1. The optimization of structures with (3-d) and (3-e) geometries failed (they led to (2-c)).

#### 3.1.2. Addition of Styrene to HStLi

The widely accepted mechanism for the anionic polymerization of styrene in non-polar solvents is that most polystyryllithium is associated into dimers and only a low proportion of non-associated polystyryllithium reacts with styrene. It is based on the experimental result that the kinetic order with respect to [PStLi] is 0.5 [6,7]. Other direct information on polymerization is the observed apparent activation energy of polymerization. Accordingly, information about the geometries and energetic values for the transition states and the reaction pathways, including the true activation energies for elementary reactions, will be helpful for understanding the detailed mechanism. In the previous section, the structure HStLi(2-c) (in which Li is coordinated to the phenyl ring) was shown to be favorable. The result of its reaction with styrene is compared with those related to HStLi(2-a) and (2-b), in which Li is coordinated to the side chain.

**Transition state.** The transition states for the addition of styrene to HStLi(2-a), (2-b), and (2-c) in Figure 2-1 were optimized, and the obtained structures are shown in Figure 4-1 and Figure 4-2. The transition states related to HStLi(2-a) and (2-b) are shown in Figure 4-1 as (4-a) through (4-c). Generally there are two structures of the transition states for each combination of styrene and HStLi, depending on the arrangement of styrene vs. HStLi (like (4-a) and (4-b) related to HStLi(2-a)). One case related to (2-b) resulted in (4-c), while the other case led to (4-a), which was originally related to (2-a). Therefore, only three cases are shown in Figure 4-1. The shortest distances between Li and the chain-end-unit carbon and between Li and styrene carbon are shown in the lower drawings. The blue arrows in each lower drawing show the main displacement vectors for the imaginary frequency of the transition state. Accordingly, the blue arrows at the α-carbon of the side chain of HStLi and the terminal carbon of the side chain of styrene indicate that these two carbon atoms react toward the direction of the arrows. The relative enthalpies (∆*Hr*), corresponding to the apparent activation energies of the reaction, and the relative free energies (∆*Gr*) for these transition states are shown in Figure 4-1. They do not differ very much from each other. Transition state (4-a) has a lower free energy than the others, and represents the addition reaction of styrene to the side-chain-coordinated HStLi hereafter.

The transition states related to HStLi(2-c) are shown in Figure 4-2 as (4-d) and (4-e), which are different in the arrangement of styrene vs. HStLi. Comparing ∆*Hr* and ∆*Gr* of the transition states (4-d) and (4-e) (in Figure 4-2) related to (2-c) with those of (4-a) through (4-c) (in Figure 4-1) related to (2-a) and (2-b), the energetic values of the former two are lower than those of the latter three by 17–18 kJ·mol^–1^. In transition state (4-a) through (4-c), Li is coordinated to the side chain of styrene (upper part) and the side chain of HStLi (lower part). In the case of (4-d) and (4-e), Li is coordinated to the phenyl ring of styrene (upper part) and the phenyl ring of HStLi (lower part). The lower energetic values of (4-d) and (4-e) compared to (4-a) through (4-c) suggest that the coordination of the phenyl rings to Li exhibit a larger interaction than that of the side chains to Li, making transition state (4-d) and (4-e) more stable. Transition state (4-d) has lower values of ∆*Hr* and ∆*Gr* than (4-e), and represents the addition reaction of styrene to the phenyl-ring-coordinated HStLi hereafter.

**Figure 5:**
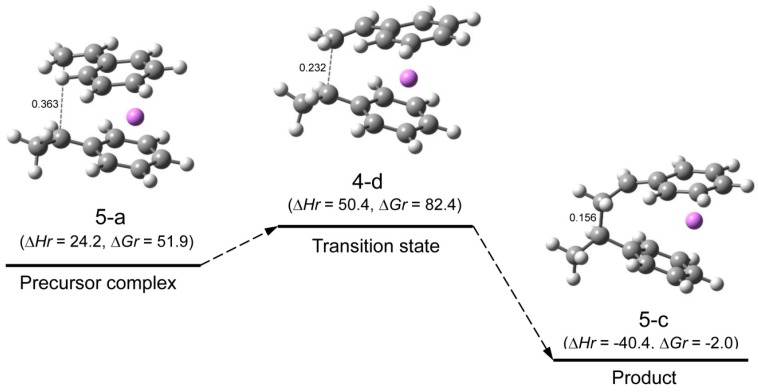
Reaction pathway for system(s-4-d) (St/[HStLi(2-c)]) in the gas phase. ∆*Hr* and ∆*Gr*, the relative enthalpy and relative free energy, are expressed in kJ·mol^–1^. Numerical values in the drawings indicate the distances between the two carbon atoms participating in the reaction, in nm.

**Figure 6:**
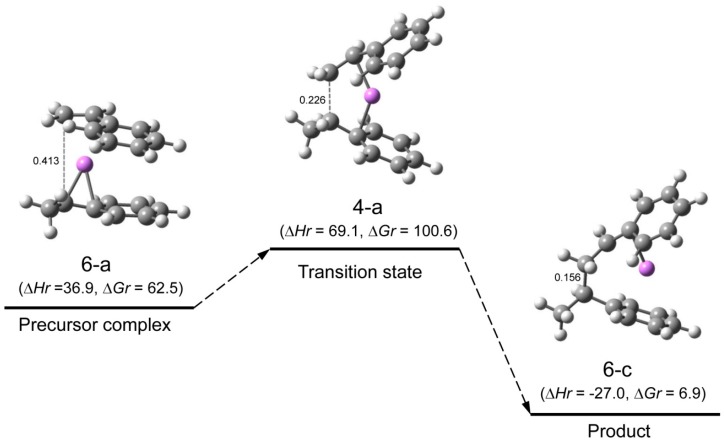
Reaction pathway for system(s-4-a) (St/[HStLi(2-a)]) in the gas phase. ∆*Hr* and ∆*Gr*, the relative enthalpy and relative free energy, are expressed in kJ·mol^–1^. Numerical values in the drawings as in Figure 5.

**Reaction pathway.** The pathway of the reaction for system St/[HStLi(2-c)] whose transition state is (4-d) (referred to as system(s-4-d) hereafter) is shown in Figure 5. The distances between the two carbon atoms participating in the reaction are shown for the precursor complex, transition state, and product. The distance of 0.363 nm for the precursor complex goes through 0.232 nm for the transition state to 0.156 nm for the product (the normal C–C single bond length). The apparent activation energy of system(s-4-d) (∆*Hr* for transition state (4-d)) is 50 kJ·mol^–1^, which consists of the enthalpy of formation for the precursor complex, 24 kJ·mol^–1^, and the true activation energy (the enthalpy from the precursor complex to the transition state), 26 kJ·mol^–1^. The pathway of the reaction for St/[HStLi(2-a)] whose transition state is (4-a) (referred to as system(s-4-a) hereafter) is shown in Figure 6. The distance between the two reacting carbon atoms for the transition state is 0.226 nm, nearly the same as that of system(s-4-d) in Figure 5, 0.232 nm. The apparent activation energy of system(s-4-a) (∆*Hr* for transition state (4-a)) is 69 kJ·mol^–1^, which consists of the enthalpy of formation for the precursor complex (37 kJ·mol^–1^) and the true activation energy (32 kJ·mol^–1^). Comparing these values with those of system(s-4-d), it is seen that the lower value for the apparent activation energy of (4-d) arises from the lower values of both the enthalpy of formation for the precursor complex and the true activation energy. Product(5-c) and (6-c) can also be expressed as HSt_2_Li whose penultimate styrene units coordinate to the Li atoms. It will be discussed in detail in the next section.

**Figure 7:**
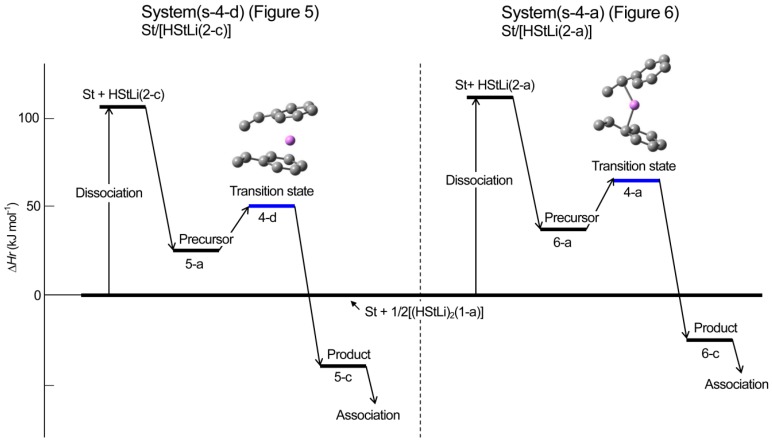
Enthalpy changes for the addition of styrene to HStLi in the gas phase.

**Figure 8:**
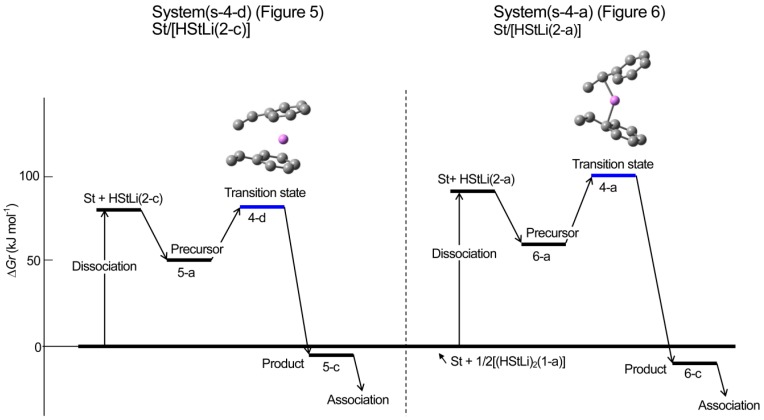
Free energy changes for the addition of styrene to HStLi in the gas phase.

Changes in the values of ∆*Hr* and ∆*Gr* of system(s-4-d) and (s-4-a) for the dissociation of (HStLi)_2_ into HStLi and the addition of styrene to non-associated HStLi are schematically shown in Figure 7 and Figure 8. These figures clearly show that system(s-4-d) related to HStLi(2-c), in which Li is coordinated to the phenyl ring, is energetically advantageous over system(s-4-a) related to HStLi(2-a), in which Li is coordinated to the side chain. In the figures the route, on which the dimer (HStLi)_2_ is first dissociated and the dissociated HStLi complexes with styrene and forms the precursor, is shown. However, the route, on which the dimer (HStLi)_2_ complexes with styrene during or before dissociation and then dissociates into the precursor, may be energetically favorable. It will be discussed in detail in Section 3.3.1.

### 3.2. (HSt_2_Li)_2_, HSt_2_Li, and the Addition of Styrene to HSt_2_Li in the Gas Phase

#### 3.2.1. (HSt_2_Li)_2_ and HSt_2_Li

One of the authors has found that the addition of styrene to RSt_m_Li (*R* = alkyl group of the initiator RLi, *m* ≥ 2) is slower than the addition to RStLi. This difference was attributed to the effect of the penultimate styrene unit [20]. Here, the behavior of (HSt_2_Li)_2_ and HSt_2_Li (with and without the penultimate unit coordination) are investigated as the first stage of the study of the penultimate unit effect.

**Figure 9:**
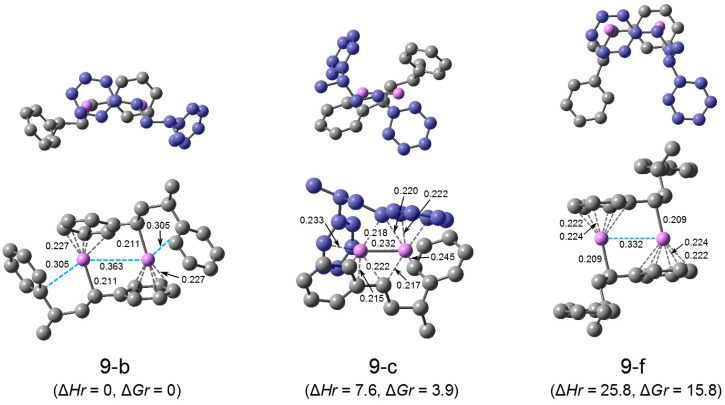
Optimized geometries and relative energies of representative (HSt_2_Li)_2_ structures with and without the penultimate unit coordination in the gas phase. The small drawing on the upper part of each structure is the overhead view of the lower drawing; the carbon atoms of one HSt_2_Li (the upper HSt_2_Li in the lower drawing) are colored in blue. For the lower drawings of (9-c), the carbon atoms of the upper HSt_2_Li are also colored in blue for clarification. Hydrogen atoms are not shown. C–Li distances less than 0.225 nm and the distances between Li and the nearest penultimate-unit carbon are shown in the lower drawings. ∆*Hr* and ∆*Gr*, the relative enthalpy and relative free energy, are expressed in kJ·mol^–1^.

**(HSt_2_Li)_2_**. There were two types of structures for (HStLi)_2_, the ‘sandwich’ type and the 6-membered-cycle type, as shown in Figure 1-1. For (HSt_2_Li)_2_, there are three types of structures, the ‘sandwich’ type with the penultimate unit coordination, the 4-membered-cycle type with the penultimate unit coordination, and structures without the penultimate unit coordination (‘sandwich’ type and 6-membered-cycle type). The optimization of these (HSt_2_Li)_2_ structures was performed, and important structures, that is, the structure with the lowest free energy for each type, are selected and shown in Figure 9 as (9-b), (9-c), and (9-f). (All (HStLi)_2_ structures studied are shown in Figures S1 and S2 in Supplementary Materials). Structure (9-b) is the ‘sandwich’ type with the penultimate unit coordination. Its dimer framework ((–St–Li)_2_ part) is like that of (1-b) in Figure 1-1. Each Li atom of (9-b) is η^1^-coordinated to the α-carbon of the chain-end unit (–St– part of HSt–St–Li) of one HSt_2_Li and η^5^-coordinated to the phenyl ring of the chain-end unit of the other HSt_2_Li. The penultimate styrene units are attracted to the Li atoms; the distances from Li to the nearest carbon atoms of the penultimate styrene units are 0.31 nm, and the Li–Li distance is ca. 0.36 nm which is longer than that of (HStLi)_2_(1-b), of ca. 0.33 nm. These results suggest a relatively strong interaction between the penultimate styrene unit and Li. (9-b) is C_2_-symmetric, the same as (1-b). Structure (9-c) is 4-membered-cycle type with the penultimate unit coordination. Each Li atom of (9-c) is η^1^-coordinated to the α-carbon of the chain-end unit and the phenyl ring of the penultimate styrene unit of one HSt_2_Li and η^3^-coordinated to the side chain of the chain-end unit of the other HSt_2_Li. The Li-Li distance is ca. 0.23 nm, the same as those of (HStLi)_2_(1-e) and (1-f). This structure looks more like the 4-membered-cycle type than the 6-membered-cycle type shown in Figure 1-1. Their penultimate units are closely attracted to Li; the distances from Li to the nearest carbon atoms of the penultimate styrene units are 0.23 and 0.25 nm. This suggests a very strong interaction between the penultimate styrene units and the Li atoms. The geometry of (9-c) is deformed and not symmetric. There are several structures of (HSt_2_Li)_2_ without the penultimate unit coordination that have the ‘sandwich’ type or 6-membered-cycle type dimer framework. Structure (9-f) has the lowest free energy of the studied (HSt_2_Li)_2_ structures without the penultimate unit coordination. In structure (9-f), the Li atoms are η^1^-coordinated to the α-carbon of the chain-end unit of one HSt_2_Li and η^6^-coordinated to the phenyl ring of the chain-end unit of the other HSt_2_Li. It has the dimer framework of (1-b). Its C–Li distances and Li–Li distance are essentially the same as those of (HStLi)_2_(1-b) in Figure 1-1. The penultimate styrene units are located far from the Li atoms (more than 0.6 nm apart) and no penultimate unit coordination is observed.

Structure (9-b) has the lowest free energy of the studied structures of (HSt_2_Li)_2_, as seen from Table A1. Accordingly, the values of relative enthalpy (∆*Hr*) and relative free energy (∆*Gr*) for the other dimers were calculated with respect to the enthalpy and free energy of (9-b) (*H*[(HSt_2_Li)_2_]_0_ and *G*[(HSt_2_Li)_2_]_0_), for which ∆*Hr* or ∆*Gr* = 0, and are shown in parentheses in Figure 9. ∆*Hr* and ∆*Gr* for (9-c) with the penultimate unit coordination are slightly higher than those for (9-b), by 8 and 4 kJ·mol^–1^, respectively. However, the values of ∆*Hr* and ∆*Gr* for (9-f) ((HSt_2_Li)_2_ without the penultimate unit coordination) are much higher, 26 and 16 kJ·mol^–1^, respectively. This difference in the relative energies clearly indicate the effect of coordination of the penultimate styrene units of (HSt_2_Li)_2_(9-b) to the Li atoms. Structures (9-b) and (9-c) have the advantage of increase in stability due to the coordination of the penultimate styrene units to Li on the one hand, and the disadvantage of decrease in stability owing to the steric hindrance such as distortion and repulsion caused by the close access of the penultimate units to the adjacent chain-end units of HSt_2_Li on the other hand. The above result suggests that the net effect of the penultimate unit coordination exceeds that of the steric hindrance.

In Section 3.1.1, ∆*Hr* and ∆*Gr* for (HStLi)_2_(1-e) and (1-f) that have 6-membered cycles were shown to be higher than those for the ‘sandwich’ type (HStLi)_2_(1-a) and (1-b) by ca. 50 kJ·mol^–1^. In the case of (HSt_2_Li)_2_(9-c), the structures became the 4-membered-cycle type, and its ∆*Hr* and ∆*Gr* values do not differ very much from those of (9-b). This suggests the effect of a strong penultimate unit coordination for (9-c). In fact, the distances between the Li atoms and the penultimate styrene units of (9-c) (0.23 and 0.25 nm) are shorter than those of (9-b) (0.31 nm), suggesting a stronger interaction of the former.

C. Z. Carlin et al. studied the in situ reaction of *sec*-butyllithium with styrene using the rapid injection NMR technique [38]. They followed the chemical shift for hydrogens of the phenyl rings of the polystyryllithium during polymerization, and attributed the change of the shift to coordination of the produced penultimate styrene unit of the polystyryllithium dimer to Li. They explained the phenomenon using a model structure of polystyryllithiium dimer with the penultimate unit coordination like (9-b) in Figure 9, which was obtained by means of the semi-empirical PM3 method.

**Figure 10:**
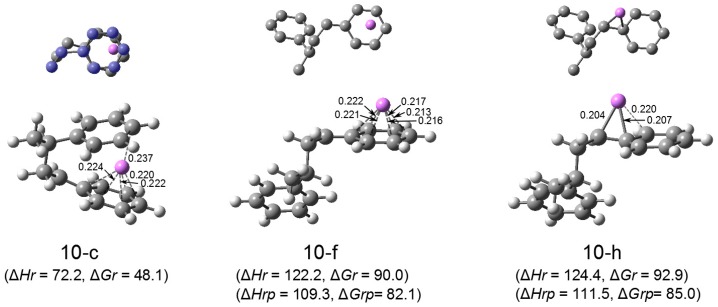
Optimized geometries and relative energies of representative HSt_2_Li structures with and without the penultimate unit coordination in the gas phase. The small drawing on the upper part of each structure is the simplified overhead view of the lower drawing; for (10-c), the carbon atoms of the penultimate styrene unit are colored in blue. C–Li distances less than 0.225 nm, and the distance between Li and the nearest penultimate-unit carbon for (10-c) are shown in the lower drawings. ∆*Hr* and ∆*Gr*, the relative enthalpy and relative free energy, are expressed in kJ·mol^–1^. ∆*Hrp* and ∆*Grp* in the lower parentheses for (10-f) and (10-h) are the enthalpy and free energy with respect to 1/2[(HSt_2_Li)_2_(9-f)], in kJ·mol^–1^.

**HSt_2_Li**. There are three types of HSt_2_Li, the structure with the penultimate unit coordination (the reaction product of HStLi with styrene), the phenyl-ring-coordinated HSt_2_Li without the penultimate unit coordination (like (2-c) in Figure 2-1), and the side-chain-coordinated HSt_2_Li without the penultimate unit coordination (like (2-a) in Figure 2-1). The optimization of these structures was performed, and the important structures of HSt_2_Li, that is, the structure with the lowest free energy for each type, are selected and shown in Figure 10 as (10-c), (10-f) and (10-h). (All HSt_2_Li structures studied are shown in Figures S3 and S4 in Supplementary Materials). As referred to in the discussion of the reaction pathways of St/HStLi in the previous section (pages 9-10), the products of the addition of styrene to HStLi become HSt_2_Li with the penultimate unit coordination. This is because styrene and the chain-end unit of HStLi are both coordinated to the Li atom on opposite sides like a ‘sandwich’ during polymerization and the final product maintains its form, as shown in Figure 5 and Figure 6. Structure (10-c) is the product of the reaction of styrene with HStLi(2-c) in Figure 2-1. It corresponds to the product whose transition state is (4-d) in Figure 4-2, and is the same as (5-c) in Figure 5, although shown here upside down and as a mirror image. The Li atom of (10-c) is η^5^-coordinated to the phenyl ring of the chain-end unit (lower part) and η^1^-coordinated to the phenyl ring of the penultimate unit (upper part). The Li atom of (10-f) is η^6^-coordinated to the phenyl ring of the chain-end unit like (2-c) in Figure 2-1. The Li atom of (10-h) is η^3^-coordinated to the side chain of the chain-end unit like (2-a) in Figure 2-1. The penultimate styrene units of (10-f) and (10-h) are located far from the Li atoms (more than 0.6 nm apart) and no penultimate unit coordination is observed.

Comparing the relative enthalpies (∆*Hr*) and relative free energies (∆*Gr*) for these three structures, (10-c) is seen to be highly stabilized due to the coordination of the penultimate styrene unit to Li. The distance between Li and the penultimate styrene unit (upper part of the drawing) of (10-c) is 0.24 nm, suggesting a strong interaction between them. ∆*Hr* and ∆*Gr* for (10-c) (72 and 48 kJ·mol^–1^), which indicate the enthalpy and free energy of the dissociation of 1/2[(HSt_2_Li)_2_(9-b)] into HSt_2_Li(10-c), are much lower than those of 1/2[(HStLi)_2_(1-a)] into HStLi(2-c) (107 and 82 kJ·mol^–1^) shown in Figure 2-1. This energetic difference is due to the coordination of the penultimate styrene unit of HSt_2_Li(10-c) to Li. Comparing ∆*Hr* and ∆*Gr* for (10-f) with those for (10-h), the energetic advantage of the structure with the phenyl-ring-type coordination, (10-f), over the structure with the side-chain-type coordination, (10-h), is again shown.

The values in the lower parentheses for (10-f) and (10-h), ∆*Hrp* and *∆Grp*, are the enthalpy and free energy for the dissociation with respect to 1/2[(HSt_2_Li)_2_(9-f)], which indicate the dissociation energies of (HSt_2_Li)_2_ without the penultimate unit coordination into HSt_2_Li without the penultimate unit coordination, as shown below for HSt_2_Li(10-f) (p.u.c.: penultimate unit coordination).

1/2[(HSt_2_Li)_2_(9-f) without p.u.c.] → [HSt_2_Li(10-f) wihout p.u.c.]
(∆*Hrp* = 109.3 kJ·mol^–1^, ∆*Grp* =82.1 kJ·mol^–1^)

Therefore, they should correspond to those for the dissociation of (HStLi)_2_ into HStLi in Figure 2-1. The values of ∆*Hrp* and ∆*Grp* for (10-f) agree with those of ∆*Hr* and ∆*Gr* for (2-c). ∆*Hrp* and ∆*Grp* for (10-h) agree approximately with ∆*Hr* and ∆*Gr* for (2-a). The geometries of the chain-ends of HSt_2_Li(10-f) and (10-h) are also essentially the same as those of the corresponding HStLi.

For dissociation of (HSt_2_Li)_2_ with the penultimate unit coordination into HSt_2_Li without the penultimate unit coordination, transformation of the HSt_2_Li structure from the state with the penultimate unit coordination to the state without the penultimate unit coordination is necessary. This is accomplished in the dimeric state (e.g., from the (9-b) type to the (9-f) type in Figure 9) or in the monomeric state of HSt_2_Li (e.g., from (10-c) to (10-f) in Figure 10) by means of rotation of the C-C single bonds of HSt_2_Li at relatively low rotation energies.

#### 3.2.2. Addition of Styrene to HSt_2_Li

In the previous section, it was shown that the relative energies for HSt_2_Li with the penultimate unit coordination were lower than those for HSt_2_Li without the penultimate unit coordination. Here the transition states for the reaction of these HSt_2_Li (with and without the penultimate unit coordination) with styrene are optimized and their energetic values are compared. The grounds for the penultimate unit effect are also discussed based on the calculated energetic values and geometries of the relevant transition states, HSt_2_Li, and (HSt_2_Li)_2_.

**Figure 11:**
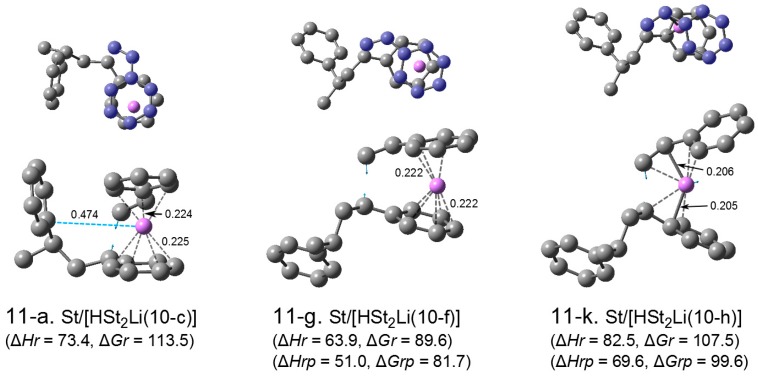
Representative transition states for the addition of styrene to HSt_2_Li with and without the penultimate unit coordination in the gas phase. In the lower drawings the shortest distances between Li and the chain-end-unit carbon, between Li and styrene carbon, and between Li and the penultimate-unit carbon for (11-a) are shown. ∆*Hr* and ∆*Gr*, the relative enthalpy and relative free energy, are expressed in kJ·mol^–1^. ∆*Hrp* and ∆*Grp* in the lower parentheses of (11-g) and (11-k) are the enthalpy and free energy with respect to (St + 1/2[(HSt_2_Li)_2_(9-f)]), in kJ·mol^–1^. Hydrogen atoms are not shown. The other drawing details as in Figure 4-1.

**Transition state.** There were three types of (HSt_2_Li)_2_ with and without the penultimate unit coordination as discussed in the previous section. The addition of styrene to these three types of HSt_2_Li were optimized, and important structures, that is, the structures with the lowest free energy for each type, are selected and shown in Figure 11 as (11-a), (11-g), and (11-k). (All transition states studied are shown in Figures S5, S6, and S7 in Supplementary Materials). Structures (11-a), (11-g), and (11-k) are the transition states of the addition of styrene to HSt_2_Li(10-c), (10-f), and (10-h) shown in Figure 10, respectively. Structure (11-a) is the transition state of St/[HSt_2_Li(10-c) with the penultimate unit coordination]. Its Li atom is η^4^-coordinated to the phenyl ring of the chain-end unit of HSt_2_Li (lower right) and η^3^–coordinated to the phenyl ring of styrene (upper right), and the penultimate styrene unit (left) is located apart from Li by ca. 0.47 nm, suggesting a weak interaction between them. Structure (11-g) is the transition state of St/[HSt_2_Li(10-f), the phenyl-ring-coordinated structure without the penultimate unit coordination]. Its Li atom is η^5^-coordinated to the phenyl ring of the chain-end unit of HSt_2_Li (lower part) and η^3^–coordinated to the phenyl ring of styrene (upper part), and the penultimate styrene unit is located far from the Li atom (more than 0.6 nm apart) and no penultimate unit coordination is observed. Structure (11-k) is the transition state of St/[HSt_2_Li(10-h), the side-chain-coordinated structure without the penultimate unit coordination]. Its Li atom is η^3^-coordinated to the side chain of the chain-end unit of HSt_2_Li (lower part) and η^4^-coordinated to the side chain of styrene (upper part), and the penultimate styrene unit is located far from the Li atom (more than 0.6 nm apart).

The values of ∆*Hr* and ∆*Gr*, which indicate the reaction barrier of HSt_2_Li with styrene, are shown in Figure 11. The energetic values for (11-a), (the transition state related to the penultimate-unit-coordinated HSt_2_Li (10-c)), are compared to those for (11-g), (the transition state related to the phenyl-ring-coordinated HSt_2_Li without the penultimate unit coordination (10-f)), and those for (11-k), (the transition state related to the side-chain-coordinated HSt_2_Li without the penultimate unit coordination (10-h)); as seen from the above ∆*Gr* values in Figure 11, (11-g) is the most stable, and although ∆*Hr* for (11-k) is higher than that for (11-a), its ∆*Gr* is lower than that for (11-a), indicating that (11-k) is more stable than (11-a). The large ∆*Gr* value for (11-a) is attributed to the steric hindrance such as repulsion and distortion caused by the close access of the penultimate styrene unit (HSt–St–Li) to the adjacent chain-end unit (HSt–St–Li) and styrene. Thus, transition state (11-g) is the most stable, followed by (11-k) and (11-a). As seen from the energetic values of ∆*Hr* and ∆*G* for (11-g) and (11-k), transition state (11-g) related to the phenyl-ring-coordinated HSt_2_Li is much more stable than the transition state (11-k) related to the side-chain-coordinated HSt_2_Li. This is as is the case for St/HStLi shown in Figure 4-2 and Figure 4-1.

Transition state (11-g), the most stable of the St/HSt_2_Li transition states, is compared to transition state (4-d) in Figure 4-2, the most stable of St/HStLi. The ∆*Hr* and ∆*G* vales for (11-g) (64 and 90 kJ·mol^–1^, respectively) are higher than those for (4-d) (50 and 82 kJ·mol^–1^, respectively) by 14 and 8 kJ·mol^–1^, indicating that the addition of styrene to HSt_2_Li is slower than to HStLi. One of the authors has already reported experimental results that the addition of styrene to polystyryllithium (RSt_m_Li, where *R* = alkyl group of the initiator and *m* ≥ 2) is slower than the addition to RStLi in cyclohexane, and attributed it to the penultimate unit effect [20]. Thus, the experimentally observed penultimate unit effect has been supported here by the quantum-chemical approach.

∆*Hrp* and ∆*Grp*, which are the enthalpy and free energy for the addition of styrene to HSt_2_Li without the penultimate unit coordination with respect to (St + 1/2[(HSt_2_Li)_2_(9-f) without the penultimate unit coordination]), are expected to be essentially the same as those for the addition of styrene to HStLi. ∆*Hrp* and ∆*Grp* for (11-g) and (11-k) are shown in the lower parentheses in Figure 11. The values of ∆*Hrp* and ∆*Grp* for (11-g) agree well with ∆*Hr* and ∆*Gr* for (4-d) in Figure 4-2. Also, ∆*Hrp* and ∆*Grp* for (11-k) agree with ∆*Hr* and ∆*Gr* for (4-a) in Figure 4-1. The geometries of the reaction area (the part of the structure exclusive of the penultimate styrene unit) of the above St/HSt_2_Li transition states are essentially the same as those of the corresponding St/HStLi transition states.

**Figure 12:**
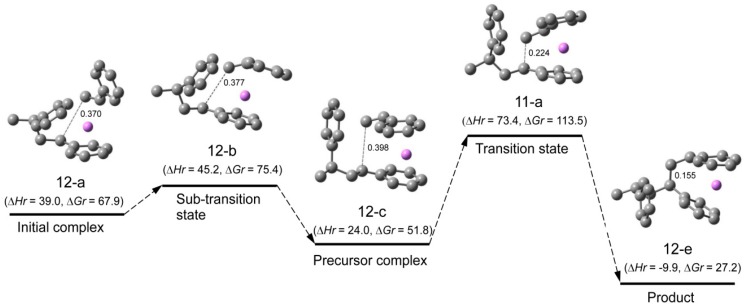
Reaction pathway for system(s-11-a) (St/[HSt_2_Li(10-c)]) in the gas phase. ∆*Hr* and ∆*Gr*, the relative enthalpy and relative free energy, are expressed in kJ·mol^–1^. Hydrogen atoms are not shown. Numerical values in the drawings as in Figure 5.

**Figure 13:**
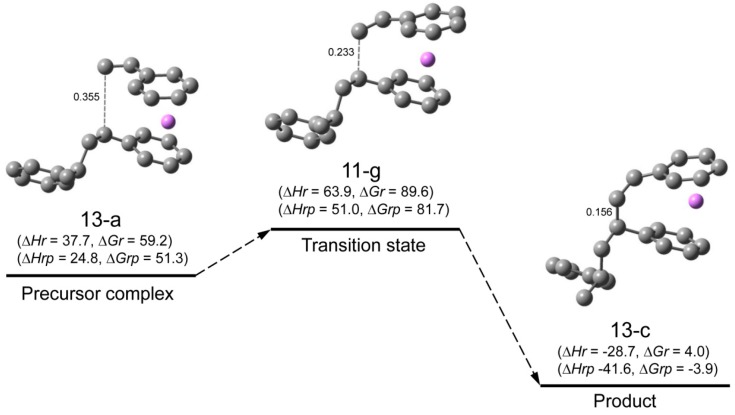
Reaction pathway for system(s-11-g) (St/[HSt_2_Li(10-f)]) in the gas phase. ∆*Hr* and ∆*Gr*, the relative enthalpy and relative free energy, are expressed in kJ·mol^–1^. ∆*Hrp* and ∆*Grp* in the lower parentheses are the enthalpy and free energy with respect to (St + 1/2[(HSt_2_Li)_2_(9-f)]), in kJ·mol^–1^. The other drawing details as in Figure 12.

**Reaction pathway.** The reaction pathway of the system St/[HSt_2_Li(10-c)] whose transition state is (11-a) (referred to as system(s-11-a) hereafter) is shown in Figure 12. The reaction proceeds in two steps. First, the initial complex is formed, which is transformed to the precursor complex through the sub-transition state. Then the addition reaction proceeds; the apparent activation energy of system(s-11-a), ∆*Hr* for transition state (11-a), is 73 kJ·mol^–1^, which consists of the enthalpy of formation for the precursor complex, 24 kJ·mol^–1^, and the true activation energy, 49 kJ·mol^–1^. The reaction pathway of system(s-11-g) (St/[HSt_2_Li(10-f)]) whose transition state is (11-g) is shown in Figure 13. The apparent activation energy of system(s-11-g), ∆*Hr* for transition state (11-g), is 64 kJ·mol^–1^, which consists of the enthalpy of formation for the precursor complex, 38 kJ·mol^–1^, and the true activation energy, 26 kJ·mol^–1^. Comparing these data, it is seen that in the case of system(s-11-a) the enthalpy of formation for the precursor complex is lower than that of system(s-11-g) due to the penultimate unit coordination of HSt_2_Li(10-c). However, the true activation energy for the former is much higher than that for the latter because of the steric hindrance caused by the close access of the penultimate styrene unit to the adjacent chain-end unit and styrene at the transition state. Eventually, the apparent activation energy of system(s-11-a) becomes higher than that of system(s-11-g).

∆*Hrp* and ∆*Grp*, the enthalpies and free energies for system(s-11-g) with respect to (St + 1/2[(HSt_2_Li)_2_(9-f) without the penultimate unit coordination]), are shown in the lower parentheses in Figure 13. ∆*Hrp* and ∆*Grp* for the precursor complex, the transition state, and the product are in good agreement with ∆*Hr* and ∆*Gr* for the corresponding structures of system(s-4-d) in Figure 5, as expected. In addition, the geometries of the reaction area of (13-a), (11-g), and (13-c) are essentially the same as the corresponding geometries in Figure 5. These results indicate that the penultimate unit effect arises from the energy difference between (HSt_2_Li)_2_(9-b) with the penultimate unit coordination and (HSt_2_Li)_2_(9-f) without the penultimate unit coordination.

**Figure 14:**
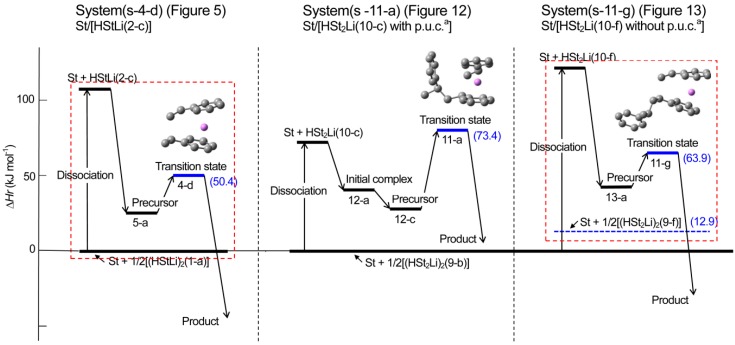
Enthalpy changes for the addition of styrene to HStLi and HSt_2_Li in the gas phase. The sub-transition state between the initial complex and the precursor for system(s-11-a) is not shown here. ^a^ p.u.c.: penultimate unit coordination.

**Figure 15:**
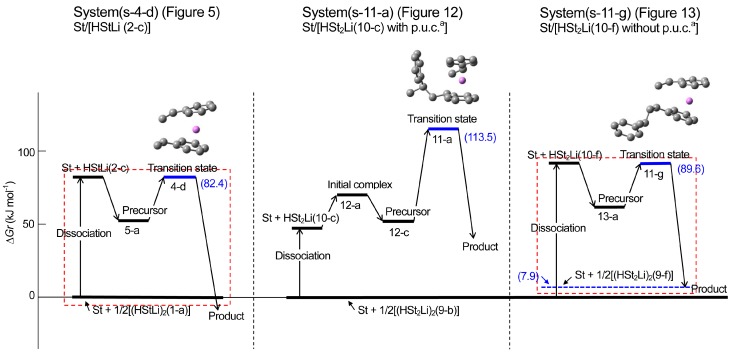
Free energy changes for the addition of styrene to HStLi and HSt_2_Li in the gas phase. The sub-transition state between the initial complex and the precursor for system(s-11-a) is not shown here. ^a^ p.u.c: penultimate unit coordination.

Changes in the values of ∆*Hr* and ∆*Gr* for system(s-11-a) and (s-11-g) are schematically shown in Figure 14 and Figure 15 along with those for system(s-4-d). The findings of the enthalpy changes that were discussed above are clearly shown in Figure 14 as discussed below.

(a) System(s-11-a) (middle part of Figure 14) vs. system(s-11-g) (right part of Figure 14)

System(s-11-a) (St/[HSt_2_Li(10-c) with the penultimate unit coordination]) gives a lower dissociation energy and enthalpy of formation for the precursor due to the penultimate unit coordination of HSt_2_Li(10-c), compared to system(s-11-g) (St/[HSt_2_Li(10-f) without the penultimate unit coordination]). However its true activation energy (from the precursor to the transition state) is much higher than that of system(s-11-g) because of the steric hindrance. Therefore, the apparent activation energy of system(s-11-a) (from the baseline to the transition state) becomes higher than that of system(s-11-g).

(b) System(s-11-g) (right part of Figure 14) vs. system(s-4-d) (left part of Figure 14)

System(s-11-g) (St/[HSt_2_Li(10-f)]) has a higher apparent activation energy (the enthalpy for the transition state) than system(s-4-d) (St/[HStLi(2-c)]), suggesting the penultimate unit effect. However, when considered with respect to (St + 1/2[(HSt_2_Li)_2_(9-f) without the penultimate unit coordination]) shown in the blue dotted line, its pattern of dissociation and reaction (inside the right dotted square) is essentially the same as that of system(s-4-d) (inside the left dotted square). It is also clearly shown that the penultimate unit effect is derived from the energy difference between (HSt_2_Li)_2_(9-f) and (HSt_2_Li)_2_(9-b) (the difference between (St + 1/2[HSt_2_Li(9-f)]) indicated by the blue dotted line and (St + 1/2[HSt_2_Li(9-b)]) indicated by the bold full line on the right side, to be exact) which is attributed to the coordination of the penultimate styrene units of (HSt_2_Li)_2_(9-b) to the Li atoms.

In Figure 15, essentially the same results are shown based on the free energy changes.

### 3.3. Solvent Effects

#### 3.3.1. Dissociation of Dimers and Addition of Styrene to HStLi and HSt_2_Li in Cyclohexane

Anionic polymerization of styrene is generally performed in polar or non-polar solvents. At an industrial scale, styrene derivatives such as solution SBR (styrene-butadiene rubber) and styrene-butadiene block copolymers have been produced in non-polar solvents. Accordingly, it is important to study the propagation reaction in non-polar solvents.

**Figure 16:**
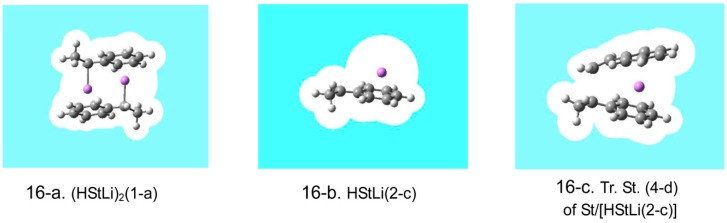
PCM models for the typical structures in solvent environment.

**Case example**. First, the behavior of the typical structures discussed in the above sections, that is, styrene, (HStLi)_2_(1-a), HStLi(2-c), and transition state (4-d) of St/[HStLi(2-c)], was studied using the PCM (polarizable continuum model) method [30,31] in the environment of the dielectric constant of cyclohexane (2.0), and the results are shown in Table 1. *H* and *G* are raw data of the enthalpy and free energy in the gas phase and in cyclohexane (originally shown in Table A1 and Table A2) expressed in units of Hartree. Comparing *H* and *G* in cyclohexane with those in the gas phase, lower values (higher absolute values) of *H* and *G* in cyclohexane compared to those in the gas phase indicate stabilization of these substances by solvation in cyclohexane. The values of solvation effect shown in the column at the far right means the difference between the enthalpy or free energy in the gas phase and that in cyclohexane, in kJ·mol^–1^. For styrene, the solvation effect is small, as expected. For the dimer (HStLi)_2_(1-a) and the transition state (4-d), the solvation effects are relatively small at 8–15 kJ·mol^–1^. However, HStLi(2-c) shows a much larger solvation effect, ca. 36 kJ·mol^–1^ of enthalpy and free energy change, indicating its increased stabilization. In Figure 16, PCM models for (HStLi)_2_(1-a), HStLi(2-c), and transition state (4-d) are shown. Figure 16 suggests that while the Li atoms of (HStLi)_2_(1-a) and transition state (4-d) are surrounded by two hydrocarbon groups such as the chain-end unit of HStLi and styrene like a ‘sandwich’ and mostly isolated from the solvent environment, a large area of the Li surface of HStLi(2-c) is directly exposed to the solvent environment and expected to be influenced much more by solvation.

**(HSt_m_Li)_2_.** Structures of the important cases discussed in the above sections were selected and optimized in the cyclohexane environment, and the results are shown in Table 2. Their relative enthalpies and relative free energies in cyclohexane, ∆*Hrch* and ∆*Grch,* were calculated in the same way as ∆*Hr* and ∆*Gr* in the gas phase. ∆*Hrch* and ∆*Grch* for the dimer (HStLi)_2_(1-c) in cyclohexane are almost the same as ∆*Hr* and ∆*Gr* in the gas phase. Firstly the Li atoms of both (HStLi)_2_(1-a) and (1-c) are surrounded by two chain-end units like a ‘sandwich’, and only small portions of the Li surface are exposed to the solvent environment; therefore, the influence of solvation is relatively small. Secondly, while the energetic values for (1-c) decrease by a small amount in cyclohexane, those for (1-a) also decrease to nearly the same extent in cyclohexane, and the solvent effect of (1-c) with respect to (1-a) becomes much smaller. The discussion on ∆*Hrch* and ∆*Grch* for (HSt_2_Li)_2_(9-f) is essentially the same as the above case, although in this case ∆*Grch* decreases a little more in cyclohexane (∆∆*G*(= ∆*Gr* − ∆*Grch*) = 8 kJ·mol^–1^).

**HSt_m_Li.** ∆*Hrch* and ∆*Grch* for the dissociation of (HStLi)_2_(1-a) into HStLi(2-c) or (2-a) and those of (HSt_2_Li)_2_(9-b) into HSt_2_Li(10-f) in cyclohexane are high (∆*Hrch* = 77–90 kJ·mol^–1^, ∆*Grch* = 51–56 kJ·mol^–1^), as shown in Table 2. However they are not so high as those in the gas phase (∆*Hr* = 107–122 kJ·mol^–1^, ∆*Gr* = 82–90 kJ·mol^–1^). In fact, ∆*Grch* for the dissociation of (HStLi)_2_(1-a) into HStLi(2-c) in cyclohexane is 53 kJ·mol^–1^, much lower than 82 kJ·mol^–1^ of ∆*Gr* in the gas phase. This decrease of ∆*Grch* in cyclohexane is due to the exposure of the large surface of Li to the solvent environment. Another important fact to be mentioned is that ∆*Grch* for the dissociation of (HStLi)_2_ into HStLi(2-a) (the side-chain-coordinated HStLi), 51 kJ·mol^–1^, is almost the same as that into (2-c) (the phenyl-ring-coordinated HStLi) in cyclohexane, 53 kJ·mol^–1^, and both are equally favorable, differing from the result in the gas phase that (2-c) is preferable.

**Reaction.** ∆*Hrch* and ∆*Grch* for the transition states and the precursor complexes of system(s-4-d), (s-4-a), and (s-11-g) in cyclohexane are also shown in Table 2. They do not differ very much from the corresponding ∆*Hr* and ∆*Gr* values in the gas phase (∆∆*H* (= ∆*Hr* − ∆*Hrch*) and ∆∆*G* are low). ∆*Hrch* and ∆*Grch* for transition state (4-d), 52 and 86 kJ·mol^–1^, are lower than those for transition state (4-a), 66 and 100 kJ·mol^–1^, by 14 kJ·mol^–1^, although the differences are a little smaller compared to those in the gas phase, 19 and 18 kJ·mol^–1^. Thus, the advantage of system(s-4-d) (the phenyl-ring-coordination type reaction) over system(s-4-a) (the side-chain-coordination type reaction) is maintained in cyclohexane, although HStLi(2-a) has almost the same enthalpy and free energy as (2-c) and both are equally favorable in cyclohexane as discussed above. The results for system(s-11-g) in cyclohexane are almost the same as those in the gas phase, and ∆*Grch* for transition state (11-g), 92 kJ·mol^–1^, is larger than that for transition state (4-d), 86 kJ·mol^–1^, indicating that the penultimate unit effect is maintained in cyclohexane.

The observed activation energies of the anionic polymerization of styrene in non-polar solvents were reported by several researchers. Worsfold et al. reported a value of 14.3 kcal·mol^–1^ (59.8 kJ·mol^–1^) in benzene [6], Ohlinger et al. reported 59.9 kJ·mol^–1^ in toluene [39], and Auguste et al. reported 75 ± 8 kJ·mol^–1^ in ethylbenzene [40], their kinetic orders all being 0.5. The calculated value of ∆Hrch for transition state (11-g) in cyclohexane (64 kJ·mol^–1^), which corresponds to the reaction barrier of addition of styrene to polystyryllithium, is in agreement with the observed apparent activation energies by Worsfold et al. and Ohlinger et al.

Some readers may have a question of how anionic polymerization proceeds without difficulty under such high dissociation energies of dimeric polystyryllithium into monomeric polystyryllithium and in such low concentrations of monomeric polystyryllithium, the question raised before by Brown for dissociation of the alkyllithium aggregates [41,42]. As for the dissociation energy, the discussion should be made based on the free energy for the polystyryllithium in solvents, instead of the enthalpy in the gas phase. The dissociation energies become smaller when it is considered. Secondly, during polymerization, styrene coexists in the system, and the complexation of styrene with monomeric and dimeric polystyryllithium should be considered. We have discussed the coordination of the penultimate styrene unit of HSt_2_Li and (HSt_2_Li)_2_ to Li in this report. It suggests the possibility of complexation of styrene with Li of the monomeric and dimeric polystyryllithium instead of coordination of the penultimate styrene unit of polystyryllithium. The path:

2St + (PStLi)_2_ → (St)_2_(PStLi)_2_ → 2[(St)(PStLi)] (precursor complex for the reaction)

will give a lower free energy than that for the dissociation of (PStLi)_2_ into PStLi, although in non-polar solvents the difference between them becomes small. The above path was also proposed by Estrin et al. who studied the dissociation of dimeric polybutadienyllithium using the ab initio calculations [43].

Some researchers assume that the polymerization proceeds through direct addition of styrene to polystyryllithium dimers [8]. For this reaction the same approach as used here for the addition of styrene to monomeric polystyryllithium is applicable, although calculations for the dimer reaction are more complicated and time-consuming. Our preliminary calculations suggest the ∆Gr value of ca. 102 kJ·mol^–1^ for the transition state of the dimer reaction in the gas phase, which is higher by ca. 20 kJ·mol^–1^ than that of the monomeric polystyryllithium reaction, 82 kJ·mol^–1^, indicating a preference for the latter reaction. Under usual polymerization conditions where concentrations of the Li initiator are very low, the difference between their rates becomes larger. It should also be mentioned that in the case of direct reaction of dimers, the kinetic order with respect to [PStLi] is 1, and not the experimentally known value of 0.5. The detailed results on the dimer reaction will be reported in our next paper in the near future.

Although the non-associated polystyryllithium is influenced appreciably by solvation in non-polar solvents, calculations in the gas phase are nevertheless useful for practical purposes. The energetic differences between organolithium compounds in the gas phase and in non-polar solvents are influenced by the extent of exposure of their Li surfaces to the solvent environment, as discussed above. Since the Li atoms of the transition states and of the dimeric polystyryllithium are surrounded by two hydrocarbon groups such as the chain-end unit and styrene like a ‘sandwich’, the relative enthalpies and relative free energies of the transition states in non-polar solvents become almost the same as those in the gas phase, and the reaction proceeds in essentially the same way as in the gas phase.

#### 3.3.2. Influence of Cyclohexane and THF Environment on (HStLi)_2_ and HStLi

In order to provide an overview of the influence of non-polar and polar solvents on the behavior of (PStLi)_2_ and PStLi, the effects of cyclohexane and THF environment on (HStLi)_2_(1-a), HStLi(2-c), and HStLi(2-a) were studied in detail.

**Figure 17:**
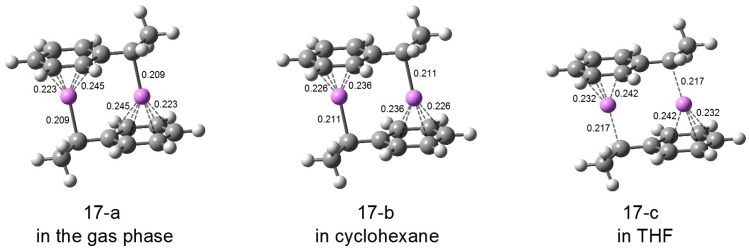
Geometries of (HStLi)_2_(1-a) in different environment. The (α)C–Li distances and the shortest and the longest distances of the (phenyl)C–Li bonds are shown in nm.

**(HStLi)_2_.** In Figure 17, the geometries of (HStLi)_2_(1-a) in the gas phase, in cyclohexane, and in THF are shown as (17-a) (which corresponds to (1-a) in Figure 1-1), (17-b), and (17-c). The values of the (α)C-Li distances and the shortest and the longest distances of the (phenyl)C-Li bonds are shown in the figure. The distances of the C-C bonds in cyclohexane and in THF are almost the same as those in the gas phase shown in Figure 1-2, and are not shown here. From Figure 17, it is clearly seen that the C-Li distances increase as the surroundings are changed from the gas phase (ε = ε_0_ = 1) to cyclohexane (ε = 2.0) and further to THF (ε = 7.4); the (α)C–Li bond becomes longer in cyclohexane, from 0.209 to 0.211 nm, and much longer in THF, 0.217 nm. While the Li atoms of (HStLi)_2_(1-a) are η^6^-coordinated to the phenyl rings in the gas phase, they are η^5^-coordinated in cyclohexane, and η^4^-coordinated in THF, and the (phenyl)C–Li distances become longer.

For calculations of atomic charges, the natural population analysis (NPA) method, which is known to give good results for lithium compounds, was used [44]. The atomic α-carbon and lithium NPA charges for (HStLi)_2_(1-a) are −0.62 and 0.66 in the gas phase, −0.62 and 0.69 in cyclohexane, and −0.63 and 0.75 in THF. The data suggest that the (α)C-Li bond in the THF environment is more ionic than in cyclohexane.

**Figure 18:**
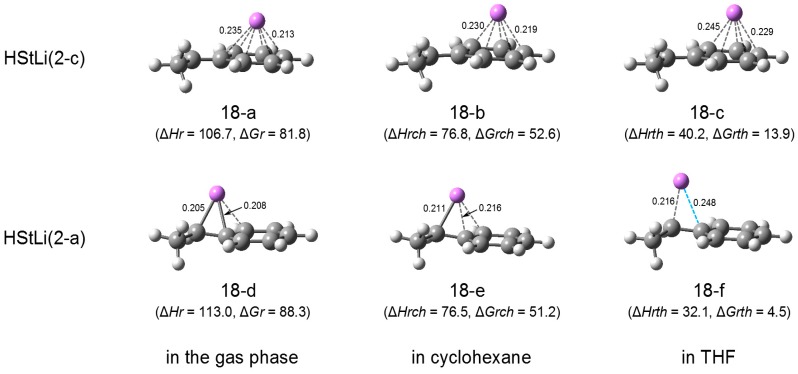
Geometries and relative energies of HStLi(2-c) ((18-a) through (18-c)) and HStLi(2-a) ((18-d) through (18-f)) in different environment. In (18-a) through (18-c), the shortest and the longest distances of the (phenyl)C–Li bonds are shown, and in (18-d) through (18-f), the distances of the (α)C–Li and (ipso)C–Li bonds are shown. ∆*Hr* and ∆*Gr*, the relative enthalpy and relative free energy in the gas phase, are expressed in kJ·mol^–1^. ∆*Hrch* and ∆*Grch* are the relative enthalpy and relative free energy in cyclohexane, and ∆*Hrth* and ∆*Grth* are those in THF, in kJ·mol^−1^.

**HStLi.** In Figure 18, the geometries of HStLi(2-c) and (2-a) are shown. Structure (18-a) (which corresponds to (2-c) in Figure 2-1), (18-b), and (18-c) are the geometries of HStLi(2-c) in the gas phase, in cyclohexane and in THF, respectively. The values of the shortest and the longest distances of the (phenyl)C–Li bonds are shown in the figure. (18-d) (which corresponds to (2-a) in Figure 2-1), (18-e), and (18-f) are the geometries of HStLi(2-a) in the gas phase, in cyclohexane and in THF, and the distances of the (α)C–Li and (ipso)C-Li bonds are shown. The C–C bond lengths of HStLi(2-c) and (2-a) in cyclohexane and in THF are almost the same as those in the gas phase shown in Figure 2-1, and are not shown here. Their C–Li distances show the same trend as those of (HStLi)_2_(1-a) discussed above. While the Li atom of HStLi(2-c) is η^6^-coordinated in the gas phase (18-a), it is η^5^-coordinated in cyclohexane (18-b) and in THF (18-c). In cyclohexane, the C–Li distances become longer than in the gas phase, and in THF they become much longer. The Li atom of HStLi(2-a) in cyclohexane (18-e) is η^3^-coordinated in the same way as in the gas phase (18-d), and the C-Li distances are longer than in the gas phase. In THF (18-f), the C–Li distances become much longer, the (ipso)C–Li distance becomes 0.248 nm, and the Li atom is η^1^-coordinated and bound only to the α-carbon of the side chain. Additionally, HStLi(2-b) in Figure 2-1 also leads to structure (18-f) when optimized in THF.

NPA charges were calculated for the α-carbon and Li atom of HStLi(2-a). The atomic α-carbon and Li NPA charges for HStLi(2-a) are –0.66 and 0.83 in the gas phase, –0.67 and 0.87 in cyclohexane, and –0.71 and 0.95 in THF, respectively. Comparing their charges with those of (HStLi)_2_(1-a) above, the (α)C–Li bonds of HStLi(2-a) are more ionic than those of (HStLi)_2_(1-a). Especially the (α)C–Li bond of HStLi(2-a) in THF is the most ionic of them. When the above charge distribution of the C-Li bond and η^1^-coordination of Li to the α-carbon of HStLi(2-a) in THF are considered, the structure of HStLi(2-a) in THF can be formulated as CH_3_–CHLi–Ph (1-phenyl-ethyllithium) or CH_3_–C^−^HLi^+^–Ph. This is the generally accepted structure in polar solvents.

∆*Hrth* and ∆*Grth* (the values of relative enthalpy and relative free energy in THF) for HStLi(2-c) and (2-a) are shown in Figure 18, along with ∆*Hr* and ∆*Gr* in the gas phase and ∆*Hrch* and ∆*Grch* in cyclohexane. They indicate the energetic values of the dissociation,

1/2[(HStLi)_2_(1-a)] → HStLi(2-c) (or (2-a))

in either the gas phase, cyclohexane, or THF. We discussed in the previous section that while the dissociation energies of (HStLi)_2_ into HStLi in the gas phase are very high and HStLi(2-c) is preferable to (2-a), those in cyclohexane are lower and HStLi(2-a) becomes as favorable as HStLi(2-c). The values of ∆*Grth* for HStLi(2-a) and (2-c) in THF (5 and 14 kJ·mol^–1^) are much lower than those for HStLi(2-a) and (2-c) in cyclohexane (51 and 53 kJ·mol^–1^), suggesting a larger solvation effect of THF. Also, the lower value of ∆*Grth* for HStLi(2-a) in THF (5 kJ·mol^–1^), compared to that for HStLi(2-c) (14 kJ·mol^–1^), indicates that HStLi(2-a) is preferable to (2-c) in THF. This result is contrary to the result in the gas phase; however, it is in agreement with what is generally accepted in polar solvents. Another important result is that ∆*Grth* for the dissociation of (HStLi)_2_(1-a) into HStLi(2-a) in THF is very low (5 kJ·mol^–1^), indicating that a considerable amount of HStLi(2-a) is in equilibrium with (HStLi)_2_(1-a). (It is known that when a small amount of THF is added to benzene solution, polystyryllithium forms 1:1 and 1:2 complexes with THF [45]. However, PCM calculations at the M062X/6-31+G(d) level indicate that in THF both HStLi and HStLi-THF complexes are present, and HStLi without complex formation is slightly preferable to HStLi-THF complexes, although in benzene solution with a small amount of THF the complexes with THF are predominant. Therefore, only the results of HStLi without complex formation are shown here).

**Figure 19:**
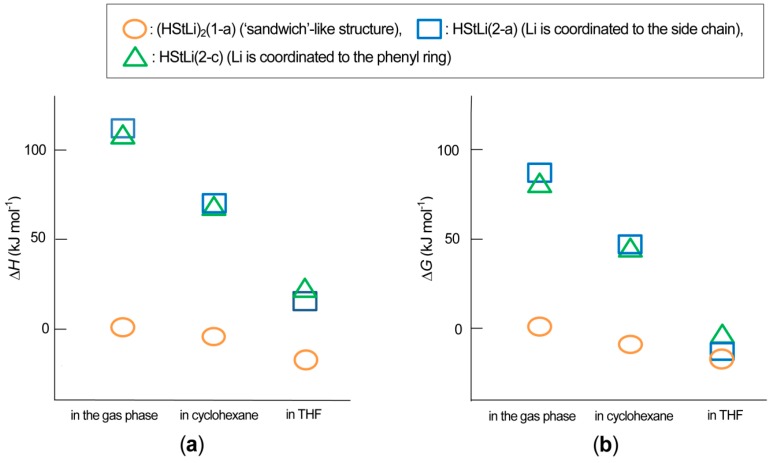
The effect of solvent environment on the enthalpies and free energies for (HStLi)_2_ and HStLi structures shown in Figure 17 and Figure 18. ∆*H* and ∆*G* were calculated with respect to 1/2[(HStLi)_2_(1-a)] in the gas phase, for which ∆*H = 0* (**a**) or ∆*G* = 0 (**b**).

**HStLi and (HStLi)_2_**. The above energetic relation is graphically shown in Figure 19. Here, for structures shown in Figure 17 and Figure 18, ∆*H* and ∆*G* were calculated with respect to 1/2(HStLi)_2_(1-a) in the gas phase, for which ∆*H* = 0 (Figure 19a) or ∆*G* = 0 (Figure 19b). The influence of the solvents on ∆*H* or ∆*G* for (HStLi)_2_(1-a), HStLi(2-c) and (2-a) can be judged by the steepness of the slopes of ∆*H* or ∆*G* in the figure. ∆*H* and ∆*G* for HStLi(2-c) and (2-a) decrease steeply as the environment is changed from the gas phase to cyclohexane and further to THF, due to the exposure of the large surface of Li to solvents. However, ∆*H* and ∆*G* for (HStLi)_2_(1-a) decrease gently with the change of environment due to its ‘sandwich’-like structure. Accordingly, the difference of ∆*G* (or ∆*H*) between HStLi(2-a) (or (2-c)) and (HStLi)_2_(1-a) that indicates the dissociation energy becomes lower and lower as the environment changes from the gas phase to cyclohexane and further to THF. Eventually the ∆*G* values of HStLi(2-c) and (2-a) are very close to that of (HStLi)_2_(1-a) in THF, as seen in Figure 19b.

In summary, in THF, the Li atom of HStLi(2-a) is bound only to the α-carbon of the side chain, HStLi(2-a) is preferable to HStLi(2-c), its (α)C-Li bond is of ionic character, and the dissociation energy of (HStLi)_2_(1-a) into HStLi(2-a) is very small. These results are in agreement with the generally accepted concepts of the behavior of monomeric polystyryllithium in polar solvents (contact ion pair) [46].

## 4. Conclusions

**Figure 20:**
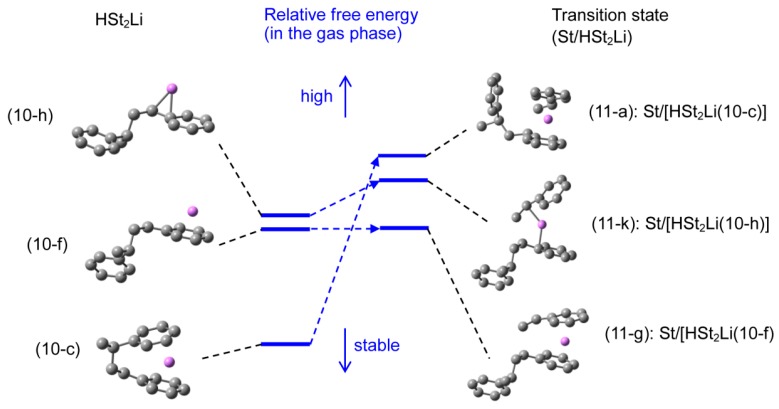
Geometries and relative free energies in the gas phase for the three types of HSt_2_Li and the transition states of their reaction with styrene.

First, a detailed process of the anionic polymerization of styrene in the gas phase is reviewed. Most of the polystyryllithium is associated into dimeric (PStLi)_2_, and a small proportion of non-associated polystyryllithium, PStLi, reacts with styrene. When polymerization proceeds and the penultimate styrene units are formed, two types of (PStLi)_2_ (with and without the penultimate unit coordination), and three types of PStLi are present. (PStLi)_2_ with the penultimate unit coordination is more stable than that without the penultimate unit coordination due to the coordination of the penultimate styrene units to Li. The three types of PStLi, which is simplified as HSt_2_Li and shown in Figure 20, are the well-known first HSt_2_Li in which the Li atom is coordinated to the side chain (10-h), the second HSt_2_Li in which Li is coordinated to the phenyl ring (10-f), both without the penultimate unit coordination, and the third HSt_2_Li in which Li is coordinated to the chain-end unit and the penultimate styrene unit (10-c). The third HSt_2_Li, (10-c), is the most stable, as expected, followed by the second HSt_2_Li, (10-f). However, the relative free energy for the transition state of the reaction of the third HSt_2_Li (10-c) with styrene (transition state (11-a)) becomes higher than those for the other two transition states, (11-g) and (11-k). This is due to the steric hindrance of (11-a) caused by the close access of the penultimate styrene unit to the adjacent chain-end unit and styrene. Transition state (11-g) related to the phenyl-ring-coordinated second HSt_2_Li (10-f) is the most stable and preferable to the other transition states, (11-a) and (11-k), suggesting that the route through (11-g) is the predominant reaction path. The transition state (11-k) related to the first HSt_2_Li (10-h) is less stable than (11-g) related to the second HSt_2_Li (10-f) because the interaction of the side chain with Li for (11-k) is smaller than that of the phenyl ring with Li for (11-g). The relative free energy for transition state (11-g), the most stable of St/HSt_2_Li transition states, is higher than that for (4-d) (Figure 4-2), the most stable transition state of the St/HStLi system. Thus, the experimentally observed penultimate unit effect that the addition of styrene to HSt_2_Li is slower than to HStLi has been demonstrated by the quantum-chemical approach. The relative energies for the transition state of St/HSt_2_Li, (11-g), with respect to (HSt_2_Li)_2_ without the penultimate unit coordination are essentially the same as those for the transition state of St/HStLi, (4-d), and the above penultimate unit effect arises from the coordination of the penultimate styrene unit of the (HSt_2_Li)_2_ dimer (one of the starting materials) to the Li atoms.

PStLi, (PStLi)_2_, and the transition states of the reaction of PStLi with styrene are stabilized in cyclohexane compared to those in the gas phase. However, the extent of stabilization differs; while PStLi is well stabilized due to the exposure of the large surface area of Li to the solvent, (PStLi)_2_ and the transition states are stabilized to a small extent because their Li atoms are surrounded by two hydrocarbon groups like a ‘sandwich’, and only small portions of the Li surface are exposed to the solvent. Therefore, the energetic values for the reaction of the St/PStLi system with respect to the starting materials (styrene and (PStLi)_2_) in cyclohexane become almost the same as those in the gas phase, and the reaction proceeds in essentially the same way as in the gas phase. The calculated reaction barrier, the relative enthalpy of the most stable transition state (11-g) in cyclohexane, is 64 kJ·mol^–1^, which is in agreement with the observed apparent activation energy in benzene of 60 kJ·mol^–1^ [6].

The above results not only support the experimentally established mechanism of the anionic polymerization of styrene in non-polar solvents, but also provide a deep insight into the structure of the reacting molecule and disclose information on the elementary processes of the reaction. Therefore, concrete and detailed discussions on the polymerization mechanism have become possible, as shown above.

In THF, the stabilization effect is much larger than in cyclohexane. The structure of the side-chain-coordinated PStLI becomes the structure in which Li is bound only to the α-carbon of the chain-end unit. It is preferable to the phenyl-ring-coordinated PStLi, its free energy of dissociation from the dimeric (PStLi)_2_ is very low, and its (α)C–Li bond is more ionic. These results are in agreement with the generally accepted concepts of the behavior of polystyryllithium in polar solvents.

In summary, the following conclusions are drawn.

(a) The widely known polymerization mechanism in non-polar solvents that most of the polystyryllithium is associated into dimers and a small proportion of non-associated polystyryllithium reacts with styrene was supported by the quantum-chemical approach. The calculated enthalpy of the reaction barrier in cyclohexane agrees with the observed apparent activation energy in benzene.
(b) The following reaction details were clarified.
  - The predominant reaction is that of styrene with the phenyl-ring-coordinated polystyryltium without the penultimate unit coordination (11-g). The reaction with the side-chain-coordinated polystyryllithium without the penultimate unit coordination (11-k) and the reaction with the polystyrllithium with the penultimate unit coordination (11-a) are less favorable.
  - The penultimate unit effect arises from the coordination of the penultimate styrene units of the polystyryllithium dimer (one of the starting materials) to the Li atoms.
(c) The reaction in non-polar solvents proceeds in essentially the same way as in the gas phase, because the Li atom of the polystyryllithium dimer and the transition state are surrounded by two hydrocarbon units like a ‘sandwich’ and their relative energies are not affected very much by the non-polar-solvent environment.
(d) In THF, the structure of the side-chain-coordinated polystyryllithium becomes the structure whose Li is bound only to the α-carbon of the chain-end unit. This PStLi is preferable to the phenyl-ring-coordinated PStLi, and its behavior is in agreement with the generally accepted concepts of polystyryllithium in polar solvents.

It has long been accepted that the Li atom is bound to the α-carbon of the active chain-end and the ionic nature of the (α)C–Li bond induces the reaction with styrene in non-polar solvents. For the above discussed case of the reaction of the phenyl-ring-coordinated active chain-end (11-g), another explanation is needed. It should also be applied consistently to the homopolymerization of butadiene and copolymerization of styrene and butadiene. However, the combinations of active chain-ends and monomers that give the lowest free energy for these cases are not simple. We speculate at present that the role of the Li atom is to stabilize the transition state by coordinating to the π-electron density of both the chain-end unit and the monomer as effectively as possible. The detailed discussion will be made in our paper on copolymerization of styrene and butadiene in the near future.

## Figures and Tables

**Figure 1-1:**
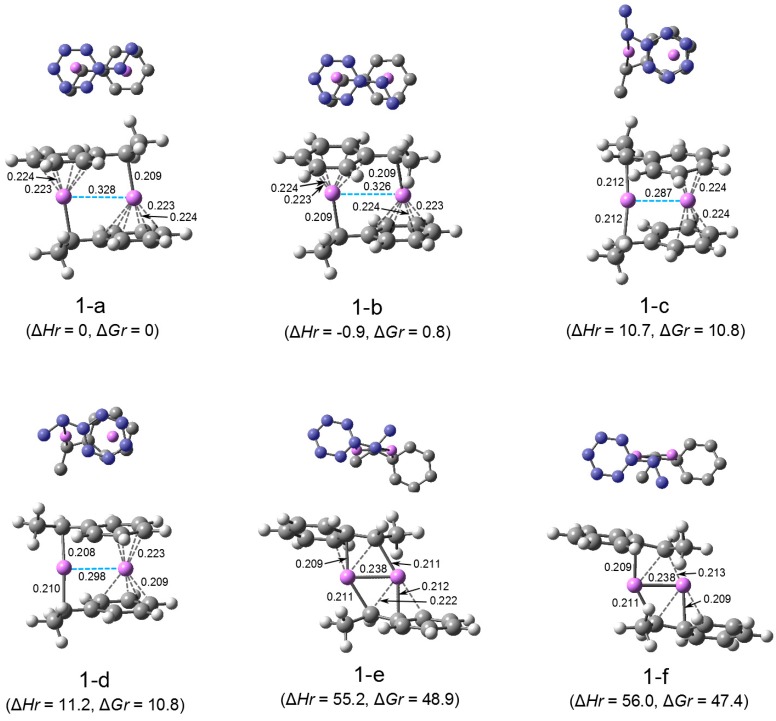
Optimized geometries and relative energies of (HStLi)_2_ in the gas phase. The small drawing on the upper part of each structure is the simplified overhead view of the lower drawing; the carbon atoms of one HStLi (the upper HStLi in the lower drawing) are colored in blue. In the lower drawings, C–Li distances less than 0.225 nm are shown. ∆*Hr* and ∆*Gr*, the relative enthalpy and relative free energy, are expressed in kJ·mol^–1^.

**Figure 1-2:**
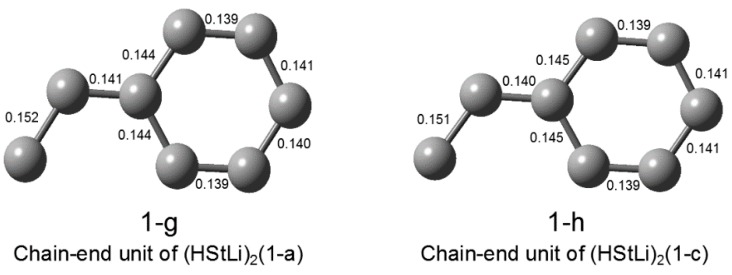
C–C bond distances of (HStLi)_2_(1-a) and (1-c). Geometry of the carbon framework of one chain-end unit (HSt-) is shown for (HStLi)_2_(1-a) and (1-c). Distances of C–C bonds are expressed in nm.

**Figure 2-1:**
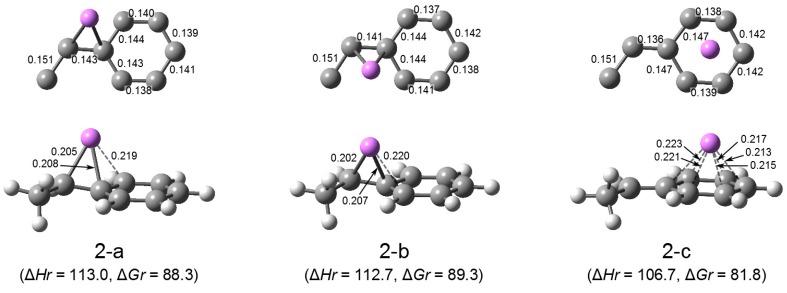
Optimized geometries and relative energies of HStLi in the gas phase. The small drawing on the upper part of each structure is the simplified overhead view of the lower drawing. C–Li distances less than 0.225 nm are shown in the lower drawings, and C–C bond distances are shown in the upper drawings. ∆*Hr* and ∆*Gr*, the relative enthalpy and relative free energy, are expressed in kJ·mol^–1^.

**Figure 2-2:**
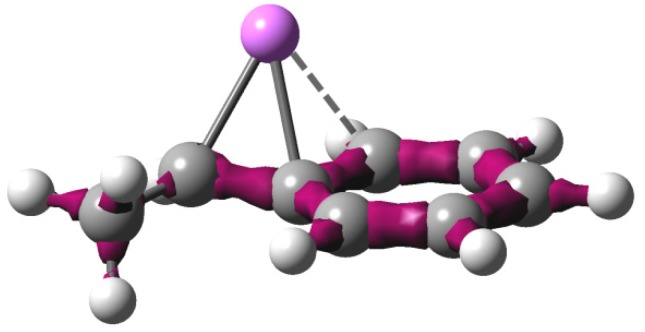
Electron density distribution of HStLi(2-a) (ea_0_^−3^ = 0.25).

**Figure 4-1:**
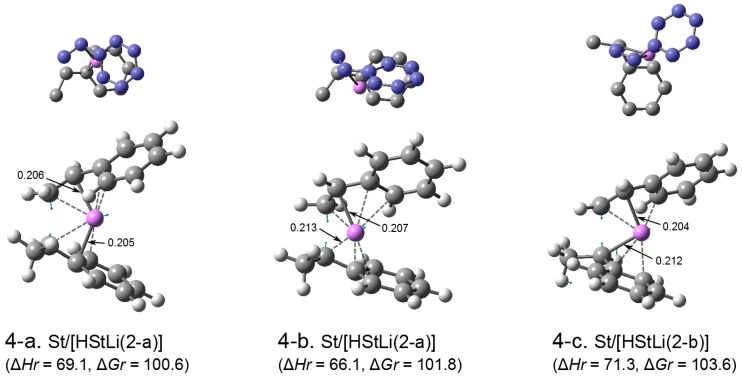
Transition states for the addition of styrene to HStLi(2-a) and (2-b) in the gas phase. The small drawing on the upper part of each structure is the simplified overhead view of the lower drawing; the carbon atoms of styrene are colored in blue. In the lower drawings, the shortest distances between Li and the chain-end-unit carbon and between Li and styrene carbon are shown. The blue arrows in the lower drawings indicate the displacement vectors for the imaginary frequency of the transition state. ∆*Hr* and ∆*Gr*, the relative enthalpy and relative free energy, are expressed in kJ·mol^–1^.

**Figure 4-2:**
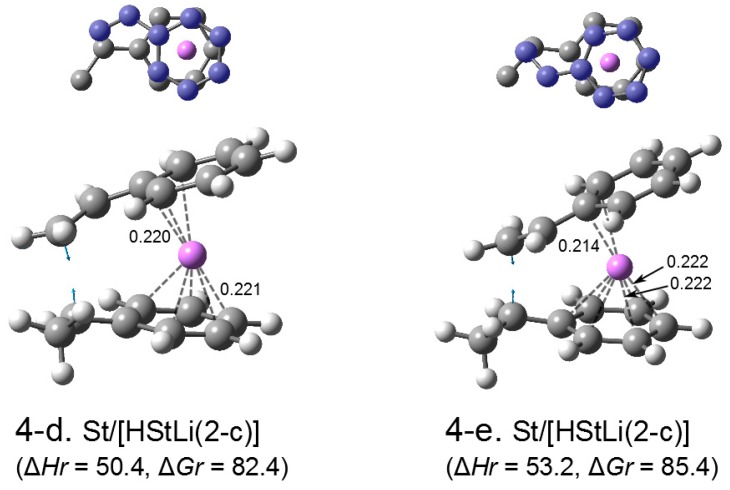
Transition states for the addition of styrene to HStLi(2-c) in the gas phase. ∆*Hr* and ∆*Gr*, the relative enthalpy and relative free energy, are expressed in kJ·mol^–1^. Drawing details as in Figure 4-1.

**Table 1:** Enthalpy and free energy changes caused by cyclohexane solvation.

	In the gas phase		In cyclohexane ^b^		Solvation effect	
	*H* ^a^	*G* ^a^	*H* ^a^	*G* ^a^	∆*H* ^c^	∆*G* ^c^
	Hartree	Hartree	Hartree	Hartree	kJ·mol^–1^	kJ·mol^–1^
Styrene	−309.367380	−309.406880	−309.368897	−309.408515	4.0	4.3
(HStLi)_2_(1-a)	−635.047732	−635.112274	−635.052605	−635.117899	12.8	14.8
HStLi(2-c)	−317.483239	−317.524976	−317.497055	−317.538903	36.3	36.6
Tr. St. (4-d) of St/HStLi(2-c)	−626.872055	–626.931624	−626.875730	−626.934819	9.6	8.4

^a^ The values of *H* and *G* are raw data of the enthalpy and free energy taken from Table A1 and Table A2; ^b^ *H* and *G* in cyclohexane were calculated using the PCM method in the environment of the dielectric constant of cyclohexane (2.0); ^c^ ∆*H* = (*H* in the gas phase) − (*H* in cyclohexane). ∆*G* = (*G* in the gas phase) − (*G* in cyclohexane).

**Table 2:** Energetic effect of the cyclohexane environment on the important structures discussed in the gas phase sections.

		In the gas phase		In cyclohexane		Solvent effect	
		∆*Hr* ^a^	∆*Gr* ^a^	∆*Hrch* ^a^	∆G*rch* ^a^	∆∆*Hr* ^b^	∆∆*Gr* ^b^
		kJ·mol^–1^	kJ·mol^–1^	kJ·mol^–1^	kJ·mol^–1^	kJ·mol^–1^	kJ·mol^–1^
**Dimer**							
(HStLi)_2_(1-a) (with respect to (HStLi)_2_(1-a))		0.0	0.0	0.0	0.0	0.0	0.0
(HStLi)_2_(1-c) (with respect to (HStLi)_2_(1-a))		10.7	10.8	9.4	10.9	1.3	−0.1
(HSt_2_Li)_2_(9-f) (with respect to (HSt_2_Li)_2_(9-b))		25.8	15.8	22.0	8.1	3.8	7.7
**Monomer (Dissociation energy)**							
HStLi(2-c) (with respect to 1/2[(HStLi)_2_(1-a)])		106.7	81.8	76.8	52.6	29.9	29.2
HStLi(2-a) (with respect to 1/2[(HStLi)_2_(1-a)])		113.0	88.3	76.5	51.2	36.5	37.1
HSt_2_Li(10-f) (with respect to 1/2[(HSt_2_Li)_2_(9-b)])		122.2	90.0	90.4	56.4	31.8	33.6
**Reaction**							
System(s-4-d) (St/[HStLi(2-c)])	Transition state (4-d)	50.4	82.4	51.1	85.7	−0.7	−3.3
	Precursor complex (5-a)	24.2	51.9	24.4	51.6	−0.2	0.3
System(s-4-a) (St/[HStLi(2-a)])	Transition state (4-a)	69.1	100.6	66.4	100.3	2.7	0.3
	Precursor complex (6-a)	36.9	62.5	34.1	60.8	2.8	1.8
System(s-11-g) (St/[HSt_2_Li(10-f)])	Transition state (11-g)	63.9	89.6	63.5	92.2	0.4	−2.6
	Precursor complex (13-a)	37.7	59.2	36.5	53.9	1.2	5.3

^a^ ∆*Hr* and ∆*Gr* are the relative enthalpy and relative free energy in the gas phase, respectively. ∆*Hrch* and ∆*Grch* are the relative enthalpy and relative free energy in cyclohexane, respectively; ^b^ ∆∆*Hr* = ∆*Hr* − ∆*Hrch*. ∆∆*Gr* = ∆*Gr* − ∆*Grch.*

## Supplementary Materials

Supplementary Materials can be found at www.mdpi.com/2073-4360/8/10/371/s1.

## Abbreviations

The following abbreviations are used in this manuscript:

IRC	Intrinsic reaction coordinate
NPA	Natural population analysis
PCM	Polarizable continuum model
PStLi	Polystyryllithium
THF	Tetrahydrofuran

## Appendix A

### Appendix A1. Calculated Energies for Studied Structures in the Gas Phase, in Cyclohexane, and in THF

**Table A1:** Calculated electronic energies, zero-point(ZP)-corrected electronic energies, enthalpies and Gibbs free energies at 25 °C in the gas phase for HStLi and HSt_2_Li, their dimers, and intermediates and products of their reaction with styrene.

Structure designation	System ^a^	State ^b^	Sym. ^c^	Electronic energy	ZP-Corrected El. energy	Enthalpy at 25 °C	Free energy at 25 °C
				Hartree	Hartree	Hartree	Hartree
	Styrene		C_s_	−309.509791	−309.375108	−309.367380	−309.406880
1-a, 17-a	(HStLi)_2_		C_i_	−635.361832	−635.066685	−635.047732	−635.112274
1-b	(HStLi)_2_		C_2_	−635.362034	−635.066958	−635.048066	−635.111985
1-c	(HStLi)_2_		C_2_	−635.357316	−635.062952	−635.043667	−635.108167
1-d	(HStLi)_2_		C_1_	−635.357150	−635.062696	−635.043457	−635.108156
1-e	(HStLi)_2_		C_1_	−635.340616	−635.045969	−635.026702	−635.093660
1-f	(HStLi)_2_		C_1_	−635.340376	−635.045695	−635.026400	−635.094205
2-a, 18-d	HStLi		C_1_	−317.636426	−317.490146	−317.480837	−317.522499
2-b	HStLi		C_1_	−317.636468	−317.490098	−317.480959	−317.522120
2-c, 18-a	HStLi		C_1_	−317.639063	−317.492539	−317.483239	−317.524976
4-a	St/HStLi(2-a)	trans. st.	C_1_	−627.164750	−626.881101	−626.864941	−626.924697
6-a	ditto	prec. com.	C_1_	−627.177240	−626.894714	−626.877210	−626.939206
6-c, 10-a	ditto	prod.	C_1_	−627.203797	−626.917733	−626.901515	−626.960375
4-b,	St/HStLi(2-a)	trans. st.	C_1_	−627.165509	−626.882034	−626.866082	−626.924258
10-b	ditto	prod.	C_1_	−627.203635	−626.917030	−626.901097	−626.959118
4-c	St/HStLi(2-b)	trans. st.	C_1_	−627.163789	−626.880182	−626.864094	−626.923495
4-d	St/HStLi(2-c)	trans. st.	C_1_	−627.171213	−626.888516	−626.872055	−626.931624
5-a	ditto	prec. com.	C_1_	−627.182144	−626.899522	−626.882012	−626.943264
5-c, 10-c	ditto	prod.	C_1_	−627.209000	−626.922552	−626.906625	−626.963788
4-e	St/HStLi(2-c)	trans. st.	C_1_	−627.170339	−626.887312	−627.870970	−626.930497
10-d	ditto	prod.	C_1_	−627.208225	−626.921535	−626.905769	−626.962507
9-a	(HSt_2_Li)_2_		C_1_	−1,254.473434	−1,253.899128	−1,253.866430	−1,253.962071
9-b	(HSt_2_Li)_2_		C_2_	−1,254.475478	−1,253.901031	−1,253.868215	−1,253.964187
9-c	(HSt_2_Li)_2_		C_1_	−1,254.472061	−1,253.898082	−1,253.865322	−1,253.962687
9-d	(HSt_2_Li)_2_		C_2_	−1,254.475433	−1,253.900563	−1,253.868171	−1,253.961955
9-e	(HSt_2_Li)_2_		C_i_	−1,254.465490	−1,253.890015	−1,253.857141	−1,253.955849
9-f	(HSt_2_Li)_2_		C_2_	−1,254.465572	−1,253.891320	−1,253.858378	−1,253.958174
10-e	HSt_2_Li		C_1_	−627.190521	−626.904064	−626.887908	−626.947895
10-f	HSt_2_Li		C_1_	−627.190042	−626.903752	−626.887554	−626.947817
10-g	HSt_2_Li		C_1_	−627.188224	−626.902301	−626.885951	−626.946715
10-h	HSt_2_Li		C_1_	−627.189002	−626.902981	−626.886712	−626.946723
11-a	St/HSt_2_Li(10-c)	trans. st.	C_1_	−936.719842	−936.296363	−936.273546	−936.345734
12-a	ditto	init. com.	C_1_	−936.733277	−936.310954	−936.286633	−936.363099
12-b	ditto	sub-trans. st.	C_1_	−936.730098	−936.307908	−936.284255	−936.360239
12-c	ditto	prec. com.	C_1_	−936.738992	−936.316726	−936.292352	−936.369237
12-e	ditto	prod.	C_1_	−936.754519	−936.328104	−936.305247	−936.378605
11-b	St/HSt_2_Li(10-c)	trans. st.	C_1_	−936.714658	−936.291484	−936.268399	−936.343249
11-c	St/HSt_2_Li(10-d)	trans. st.	C_1_	−936.716195	−936.291972	−936.269438	−936.340815
11-d	St/HSt_2_Li(10-d)	trans. st.	C_1_	−936.712791	−936.288519	−936.265940	−936.338335
11-e	St/HSt_2_Li(10-e)	trans. st.	C_1_	−936.722598	−936.300214	−936.276740	−936.354197
11-f	St/HSt_2_Li(10-e)	trans. st.	C_1_	−936.721523	−936.298977	−936.275484	−936.353934
11-g	St/HSt_2_Li(10-f)	trans. st.	C_1_	−936.722896	−936.300627	−936.277131	−936.354853
13-a	ditto	prec. com.	C_1_	−936.734032	−936.311594	−936.287128	−936.366417
13-c	ditto	prod.	C_1_	−936.761364	−936.335489	−936.312428	−936.387432
11-h	St/HSt_2_Li(10-f)	trans. st.	C_1_	−936.722064	−936.299676	−936.276150	−936.354137
11-i	St/HSt_2_Li(10-g)	trans. st.	C_1_	−936.715325	−936.292112	−936.268776	−936.347402
11-j	St/HSt_2_Li(10-g)	trans. st.	C_1_	−936.714844	−936.291764	−936.268515	−936.346813
11-k	St/HSt_2_Li(10-h)	trans. st.	C_1_	−936.716557	−936.293320	−936.270052	−936.348016
11-m	St/HSt_2_Li(10-h)	trans. st.	C_1_	−936.716907	−936.293762	−936.270585	−936.347825

^a^ ‘ditto’ here means that not only the structure of HSt_m_Li but also the arrangement of styrene vs. HSt_m_Li are the same as those of the upper column; ^b^ trans. st.: transition state, prec. com.: precursor complex, prod.: product, init. com.: initial complex, sub-trans. st.: sub-transition state; ^c^ sym.: symmetry.

**Table A2:** Calculated energies in cyclohexane for the important structures discussed in the gas phase sections.

Structure designation ^a^	System ^b^	State ^c^	Sym. ^d^	Electronic energy	ZP-corrected El. energy	Enthalpy at 25 °C	Free energy at 25 °C
				Hartree	Hartree	Hartree	Hartree
	Styrene		C_s_	−309.511320	−309.376626	−309.368897	−309.408515
[1-a], 17-b	(HStLi)_2_		C_i_	−635.366084	−635.366084	−635.052605	−635.117899
[1-c]	(HStLi)_2_		C_2_	−635.362243	−635.362243	−635.049025	−635.113755
[2-a], 18-e	HStLi		C_1_	−317.652324	−317.506730	−307.497171	−317.539434
[2-c], 18-b	HStLi		C_1_	−317.652436	−317.506489	−317.497055	−317.538903
[4-a]	St/HStLi(2-a)	trans. st.	C_1_	−627.169572	−626.986079	−626.869921	−626.929248
[6-a]	ditto	prec. com.	C_1_	−627.182145	−626.899752	−626.882229	−626.944293
[4-d]	St/HStLi(2-c)	trans. st.	C_1_	−627.174743	−626.892141	−626.875730	−626.934819
[5-a]	ditto	prec. com.	C_1_	−627.185847	−626.903557	−626.885888	−626.947806
[9-b]	(HSt_2_Li)_2_		C_2_	−1,254.480646	−1,253.906131	−1,253.873591	−1,253.967380
[9-f]	(HSt_2_Li)_2_		C_2_	−1,254.472199	−1,253.898075	−1,253.865210	−1,253.964282
[10-f]	HSt_2_Li		C_1_	−627.204399	−626.918633	−626.902355	−626.962226
[11-g]	St/HSt_2_Li(10-f)	trans. st.	C_1_	−936.727396	−936.304729	−936.281524	−936.357091
[13-a]	ditto	prec. com.	C_1_	−936.738415	−936.316355	−936.291782	−936.371686

^a^ [n-x] means the structure in cyclohexane corresponding to the structure n-x in the gas phase; ^b^ ‘ditto’ here means that not only the structure of HSt_m_Li but also the arrangement of styrene vs. HSt_m_Li are the same as those of the upper column; ^c^ trans. st.: transition state, prec. com.: precursor complex; ^d^ Sym.: Symmetry.

**Table A3:** Calculated energies in THF for (HStLi)_2_(1-a), HStLi(2-a) and (2-c).

Structure designation ^a^	System	Symmetry	Electronic energy	ZP-Corrected El. energy	Enthalpy at 25 °C	Free energy at 25 °C
			Hartree	Hartree	Hartree	Hartree
[1-a], 17-c	(HStLi)_2_	C_i_	−635.373761	−635.080347	−635.061090	−635.125151
[2-a], 18-f	HStLi	C_1_	−317.672857	−317.528007	−317.518318	−317.560874
[2-c], 18-c	HStLi	C_1_	−317.670111	−317.524826	−317.515252	−317.557286

^a^ [n-x] means the structure in THF corresponding to the structure n-x in the gas phase.

### Appendix A2. Basis Set Superposition Errors

The basis set superposition errors (BSSE) were estimated for some important structures, i.e., (HStLi)_2_(1-a) and transition state (4-d), using the counterpoise method [32]. The electronic energies of BSSE for (HStLi)_2_(1-a) and transition state (4-d) were 13.5 and 9.8 kJ·mol^–1^, respectively. However, the BSSE works in the direction of decreasing the absolute energetic values and the effect of BSSE for transition state (4-d) with respect to [St + 1/2(HStLi)_2_(1-a)] became smaller, 3.1 kJ·mol^–1^, the value that will not affect the conclusion of our discussion. Therefore, the energetic values without the BSSE correction were used for all structures studied in the present paper.
