# Supplementary material for: New Vistas on the Anionic Polymerization of Styrene in Non-Polar Solvents by Means of Density Functional Theory
Source: Polymers (Basel). 2016 Oct 20;8(10):371. doi: 10.3390/polym8100371 (PMC6431960; doi:10.3390/polym8100371)
Supplement: Supplementary file 1 [file polymers-08-00371-s001.pdf]

Supplementary Materials: New Vistas on the Anionic Polymerization of Styrene in Non-Polar Solvents by Means of Density Functional Theory

Hideo Morita and Marcel Van Beylen

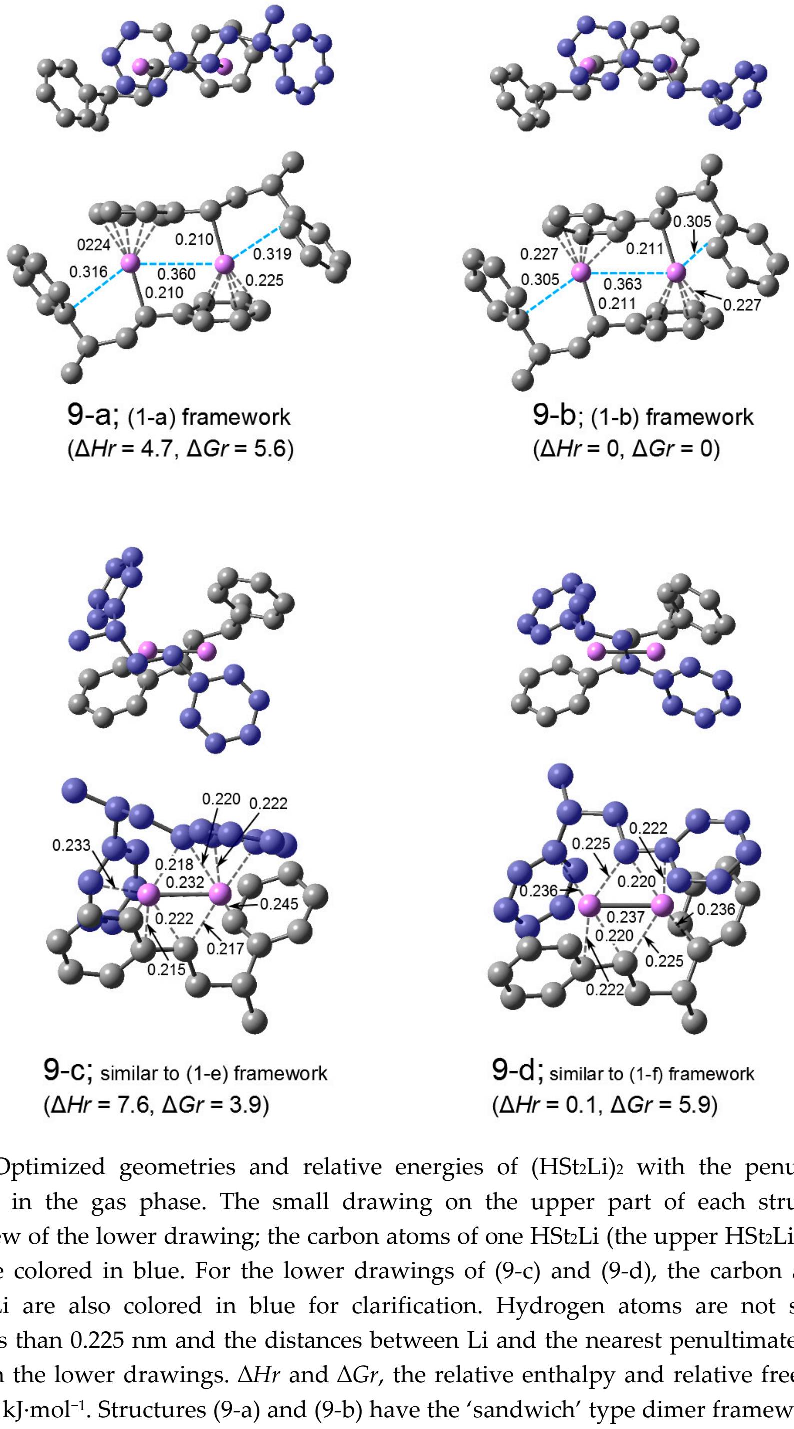

Figure S1. Optimized geometries and relative energies of $(HSt_2Li)_2$ with the penultimate unit coordination in the gas phase. The small drawing on the upper part of each structure is the overhead view of the lower drawing; the carbon atoms of one HSt_2Li (the upper HSt_2Li in the lower drawing) are colored in blue. For the lower drawings of (9-c) and (9-d), the carbon atoms of the upper HSt_2Li are also colored in blue for clarification. Hydrogen atoms are not shown. C–Li distances less than 0.225 nm and the distances between Li and the nearest penultimate-unit carbon are shown in the lower drawings. ΔHr and ΔGr, the relative enthalpy and relative free energy, are expressed in kJ·mol^{-1}. Structures (9-a) and (9-b) have the 'sandwich' type dimer framework like (1-a) and (1-b) in Figure 1-1, respectively. Structures (9-c) and (9-d) have the 4-membered-cycle type dimer framework similar to the 6-membered-cycle type (1-e) and (1-f) in Figure 1-1, respectively.

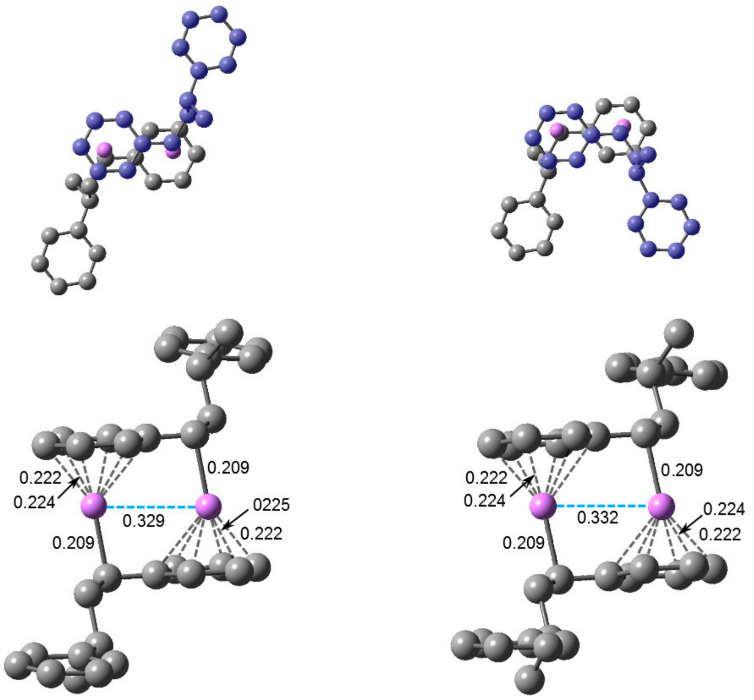

9-e; (1-a) framework
(ΔHr = 29.9, ΔGr = 21.9)

9-f; (1-b) framework
(ΔHr = 25.8, ΔGr = 15.8)

Figure S2. Optimized geometries and relative energies of $(HSt_2Li)_2$ without the penultimate unit coordination in the gas phase. The small drawing on the upper part of each structure is the overhead view of the lower drawing; the carbon atoms of one HSt_2Li (the upper HSt_2Li in the lower drawing) are colored in blue. Hydrogen atoms are not shown. C-Li distances less than 0.225 nm are shown in the lower drawings. ΔHr and ΔGr, the relative enthalpy and relative free energy, are expressed in $kJ \cdot mol^{-1}$. There are several structures without the penultimate unit coordination that have the 'sandwich' type or 6-membered-cycle type dimer framework. Structures (9-e) and (9-f) are those that have lower free energies than the others, and have the (1-a) and (1-b) dimer framework, respectively.

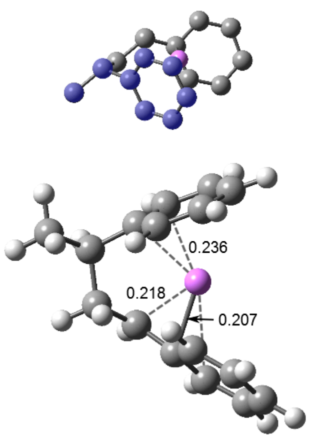

10-a; Product of St/[HStLi(2-a)]
(ΔHr = 85.6, ΔGr = 57.0)

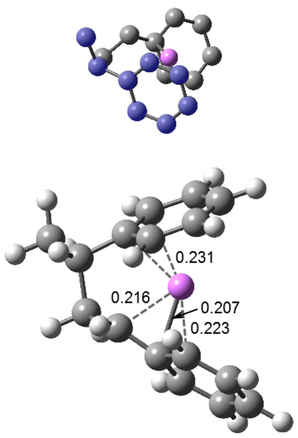

10-b; Product of St/[HStLi(2-a)]
(ΔHr = 86.7, ΔGr = 60.3)

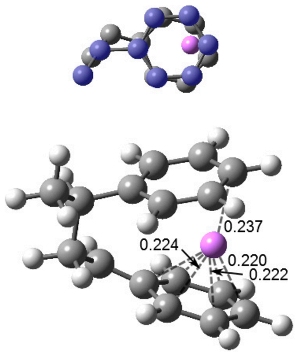

10-c; Product of St/[HStLi(2-c)]
(ΔHr = 72.2, ΔGr = 48.1)

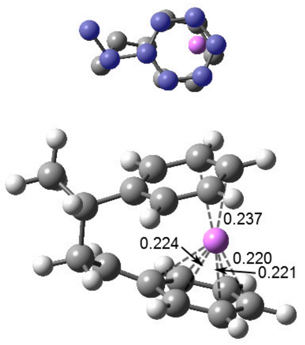

10-d; Product of St/[HStLi(2-c)]
(ΔHr = 74.4, ΔGr = 51.4)

Figure S3. Optimized geometries and relative energies of HSt_2Li with the penultimate unit coordination in the gas phase. The small drawing on the upper part of each structure is the simplified overhead view of the lower drawing; the carbon atoms of the penultimate styrene unit are colored in blue. C–Li distances less than 0.225 nm and the distance between Li and the nearest penultimate unit carbon are shown in the lower drawings. ΔHr and ΔGr, the relative enthalpy and relative free energy, are expressed in $kJ \cdot mol^{-1}$. Structures (10-a) and (10-b) are the product of the addition of styrene to HStLi(2-a) whose transition states are (4-a) and (4-b) in Figure 4-1, respectively. Structures (10-c) and (10-d) are the products of the addition of styrene to HStLi(2-c) in Figure 2-1 whose transition states are (4-d) and (4-e) in Figure 4-2, respectively. Structures (10-a) and (10-c) correspond to product (6-c) in Figure 6 and (5-c) in Figure 5, respectively, although shown here upside down and as mirror images.

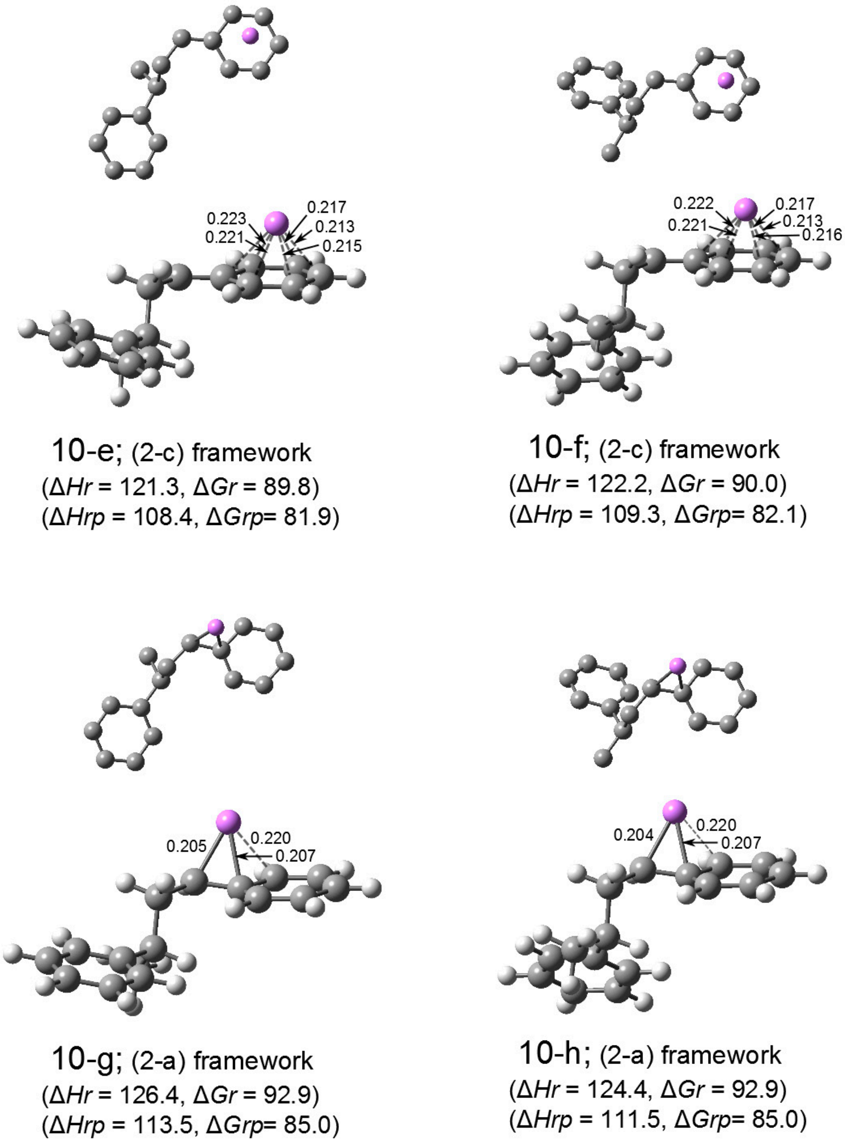

Figure S4. Optimized geometries and relative energies of HSt_2Li without the penultimate unit coordination in the gas phase. The small drawing on the upper part of each structure is the simplified overhead view of the lower drawing. C–Li distances less than 0.225 nm are shown in the lower drawings. ΔHr and ΔGr, the relative enthalpy and relative free energy, are expressed in kJ·mol^{-1}. ΔHrp and ΔGrp in the lower parentheses are the enthalpy and free energy with respect to 1/2[$(HSt_2Li)_2$(9-f)], in kJ·mol^{-1}. There are several HSt_2Li structures without the penultimate unit coordination whose Li are coordinated to the phenyl ring of the chain-end unit like HStLi(2-c) in Figure 2-1. Structures (10-e) and (10-f) are those that have lower free energies than the others. They are different in the arrangement of the methyl and phenyl groups of the penultimate unit. Also there are several HSt_2Li structures without the penultimate unit coordination whose Li are coordinated to the side chain of the chain-end unit like HStLi(2-a) in Figure 2-1. Structures (10-g) and (10-h) are those that have lower free energies than the others. They are different in the arrangement of the methyl and phenyl groups of the penultimate unit. ((10-f) was selected as the representative of the phenyl-ring-coordinated HSt_2Li in Figure 10 considering the fact that at the transition state St/HStLi(10-f) is preferable to St/HStLi(10-e) as shown in the following Figure S6 and the free energy difference between (10-e) and (10-f) is very small although (10-e) has a lower free energy than (10-f)).

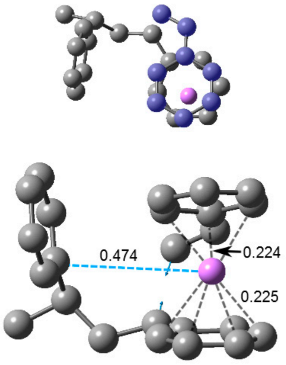

11-a. St/[HSt_2Li(10-c)]
(ΔHr = 73.4, ΔGr = 113.5)

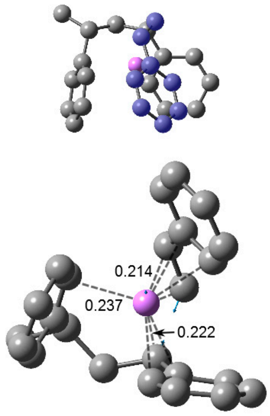

11-b. St/[HSt_2Li(10-c)]
(ΔHr = 86.9, ΔGr = 120.0)

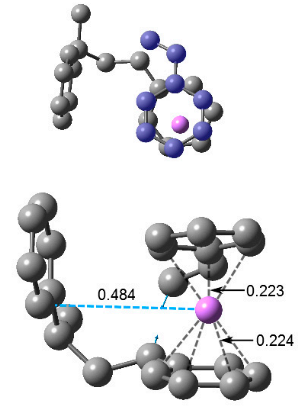

11-c. St/[HSt_2Li(10-d)]
(ΔHr = 84.1, ΔGr = 126.4)

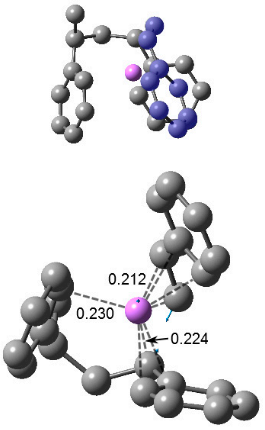

11-d. St/[HSt_2Li(10-d)]
(ΔHr = 93.3, ΔGr = 133.0)

Figure S5. Transition states for the addition of styrene to HSt_2Li with the penultimate unit coordination in the gas phase. In the lower drawing of each structure the shortest distances between Li and the chain-end-unit carbon, between Li and styrene carbon, and between Li and the penultimate-unit carbon are shown. ΔHr and ΔGr, the relative enthalpy and relative free energy, are expressed in kJ·mol^{-1}. Hydrogen atoms are not shown. The other drawing details as in Figure 4-1. Structures (11-a) and (11-b) are the transition states of the addition of styrene to HSt_2Li(10-c) shown in Figure S3. They are different in the arrangement of HSt_2Li vs. styrene. Structures (11-c) and (11-d) are the transition states of the addition of styrene to HSt_2Li(10-d) shown in Figure S3. They are different in the arrangement of HSt_2Li vs. styrene.

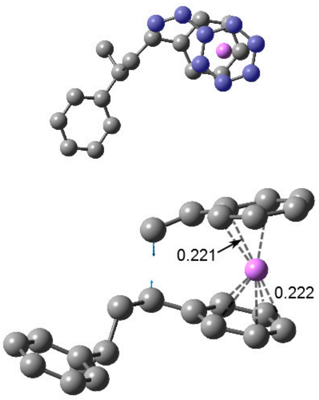

11-e. St/[HSt_2Li(10-e)]
(ΔHr = 65.0, ΔGr = 91.3)
(ΔHrp = 52.1, ΔGrp = 83.4)

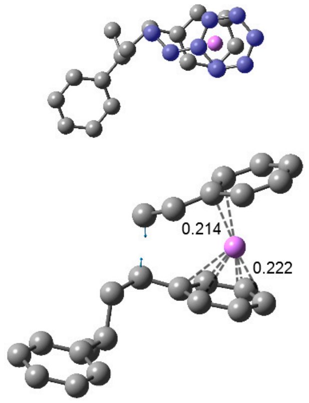

11-f. St/[HSt_2Li(10-e)]
(ΔHr = 68.3, ΔGr = 92.0)
(ΔHrp = 55.4, ΔGrp = 84.1)

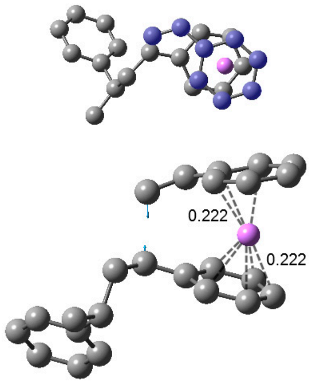

11-g. St/[HSt_2Li(10-f)]
(ΔHr = 63.9, ΔGr = 89.6)
(ΔHrp = 51.0, ΔGrp = 81.7)

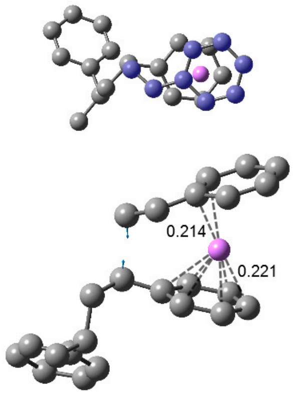

11-h. St/[HSt_2Li(10-f)]
(ΔHr = 66.5, ΔGr = 91.5)
(ΔHrp = 53.6, ΔGrp = 83.6)

Figure S6. Transition states for the addition of styrene to the phenyl-ring-coordinated HSt_2Li without the penultimate unit coordination in the gas phase. ΔHr and ΔGr, the relative enthalpy and relative free energy, are expressed in $kJ \cdot mol^{-1}$. ΔHrp and ΔGrp in the lower parentheses are the enthalpy and free energy with respect to (St + 1/2[$(HSt_2Li)_2$(9-f)]), in $kJ \cdot mol^{-1}$. Hydrogen atoms are not shown. The other drawing details as in Figure 4-1. Structures (11-e) and (11-f) are the transition states of the addition of styrene to HSt_2Li(10-e) shown in Figure S4. They are different in the arrangement of HSt_2Li vs. styrene. Structures (11-g) and (11-h) are the transition states of the addition of styrene to HSt_2Li(10-f) shown in Figure S4. They are different in the arrangement of HSt_2Li vs. styrene.

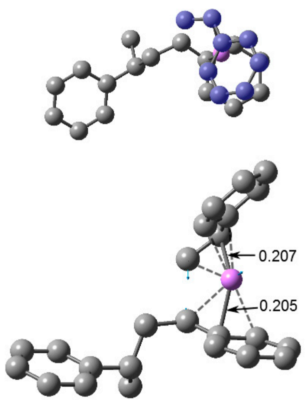

11-i. St/[HSt_2Li(10-g)]
(ΔHr = 85.9, ΔGr = 109.1)
(ΔHrp = 73.0, ΔGrp = 101.2)

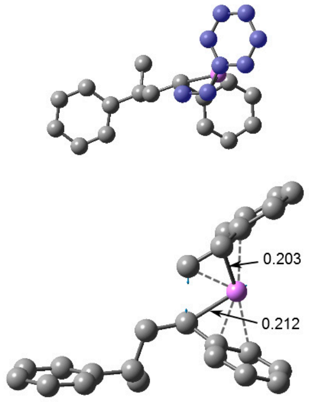

11-j. St/[HSt_2Li(10-g)]
(ΔHr = 86.6, ΔGr = 110.7)
(ΔHrp = 73.7, ΔGrp = 102.8)

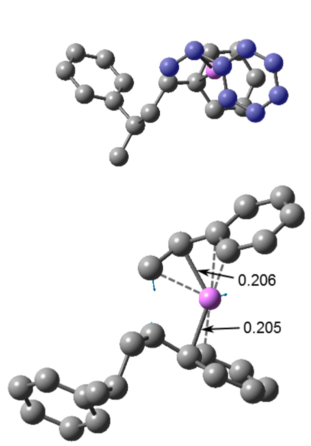

11-k. St/[HSt_2Li(10-h)]
(ΔHr = 82.5, ΔGr = 107.5)
(ΔHrp = 69.6, ΔGrp = 99.6)

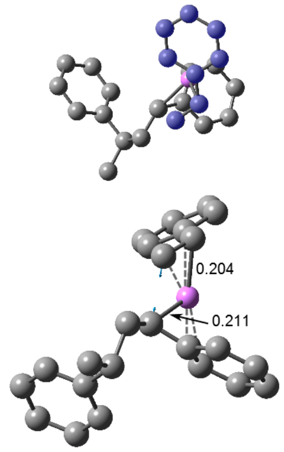

11-m. St/[HSt_2Li(10-h)]
(ΔHr = 81.1, ΔGr = 108.0)
(ΔHrp = 68.2, ΔGrp = 100.1)

Figure S7. Transition states for the addition of styrene to the side-chain-coordinated HSt_2Li without the penultimate unit coordination in the gas phase. ΔHr and ΔGr, the relative enthalpy and relative free energy, are expressed in kJ·mol^{-1}. ΔHrp and ΔGrp in the lower parentheses are the enthalpy and free energy with respect to (St + 1/2[$(HSt_2Li)_2$(9-f)]), in kJ·mol^{-1}. The other drawing details as in Figure S6. Structures (11-i) and (11-j) are the transition states of the addition of styrene to HSt_2Li(10-g) shown in Figure S4. They are different in the arrangement of HSt_2Li vs. styrene. Structures (11-k) and (11-m) are the transition states of the addition of styrene to HSt_2Li(10-h) shown in Figure S4. They are different in the arrangement of HSt_2Li vs. styrene.
